# Mitochondria‐Targeted Artificial “Nano‐RBCs” for Amplified Synergistic Cancer Phototherapy by a Single NIR Irradiation

**DOI:** 10.1002/advs.201800049

**Published:** 2018-05-21

**Authors:** Liang Zhang, Dong Wang, Ke Yang, Danli Sheng, Bin Tan, Zhigang Wang, Haitao Ran, Hengjing Yi, Yixin Zhong, Han Lin, Yu Chen

**Affiliations:** ^1^ Department of Ultrasound the First Affiliated Hospital of Chongqing Medical University Chongqing 400010 China; ^2^ Pediatric Research Institute Children's Hospital of Chongqing Medical University Chongqing 400014 China; ^3^ Institute of Ultrasound Imaging the Second Affiliated Hospital of Chongqing Medical University Chongqing 400010 China; ^4^ State Key Laboratory of High Performance Ceramics and Superfine Microstructures Shanghai Institute of Ceramics Chinese Academy of Sciences Shanghai 200050 China

**Keywords:** nanoliposomes, nanomedicine, perfluorooctyl bromide, photodynamic therapy, photothermal therapy

## Abstract

Phototherapy has emerged as a novel therapeutic modality for cancer treatment, but its low therapeutic efficacy severely hinders further extensive clinical translation and application. This study reports amplifying the phototherapeutic efficacy by constructing a near‐infrared (NIR)‐responsive multifunctional nanoplatform for synergistic cancer phototherapy by a single NIR irradiation, which can concurrently achieve mitochondria‐targeting phototherapy, synergistic photothermal therapy (PTT)/photodynamic therapy (PDT), self‐sufficient oxygen‐augmented PDT, and multiple‐imaging guidance/monitoring. Perfluorooctyl bromide based nanoliposomes are constructed for oxygen delivery into tumors, performing the functions of red blood cells (RBCs) for oxygen delivery (“Nano‐RBC” nanosystem), which can alleviate the tumor hypoxia and enhance the PDT efficacy. The mitochondria‐targeting performance for enhanced and synergistic PDT/PTT is demonstrated as assisted by nanoliposomes. In particular, these “Nano‐RBCs” can also act as the contrast agents for concurrent computed tomography, photoacoustic, and fluorescence multiple imaging, providing the potential imaging capability for phototherapeutic guidance and monitoring. This provides a novel strategy to achieve high therapeutic efficacy of phototherapy by the rational design of multifunctional nanoplatforms with the unique performances of mitochondria targeting, synergistic PDT/PTT by a single NIR irradiation (808 nm), self‐sufficient oxygen‐augmented PDT, and multiple‐imaging guidance/monitoring.

## 1 Introduction

Phototherapy, such as the well‐known photothermal therapy (PTT) and photodynamic therapy (PDT), is emerging as one of the most representative therapeutic modalities for the treatment of cancers due to its unique characteristics including noninvasiveness, high selectivity, and low systemic toxicity as compared to traditional chemotherapy and radiotherapy.^1^ PTT usually employs photothermal‐conversion agent (PTCA) to convert light into thermal energy for localized cancer hyperthermia under appropriate photoirradiation.^2^ Although PTT is effective for tumor ablation, it suffers from the possibility of tumor regrowth/reoccurrence caused by insufficient hyperthermia or uneven heat delivery within tumor region.^3^ Alternatively, PDT employs toxic reactive oxygen species (ROS) to kill cancer cells, which are produced by photo excitation toward PTCA.^4^ The dissolved oxygen (O_2_) in surrounding microenvironment of tumor is involved to produce toxic singlet oxygen (^1^O_2_) and cause the irreversible damage of tumor cell/tissue afterward.^5^ However, some intrinsic barriers such as the hypoxia nature of solid tumor and instantaneous short lifetime of ROS cause the low production efficiency of ^1^O_2_ and therefore decrease the therapeutic outcome of PDT.^6^ It is highly expected that the rational combination of PTT and PDT could cause the synergistically therapeutic efficacy/outcome based on their both photoinduced nature for cancer therapy.^7^ However, it is still highly challenging to achieve the desirable high efficiency and selectivity of these two combined phototherapies.

Mitochondrion, as a vital cellular organelle, plays key roles in energy production and programed cell death such as apoptosis.^8^ Especially, mitochondria have been recognized as an attractive subcellular target to improve the therapeutic efficacy on combating cancer.^9^ It is noted that some researchers had achieved enhanced PDT efficacy upon laser irradiation by developing a mitochondria‐targeting photosensitizer‐triphenylphosphine‐based nanosystem.^10^ In addition, it has also been verified that mitochondria is susceptible to hyperthermia, based on which diverse nanoplatforms have been designed for mitochondria‐targeting PTT by modifying these nanosystems with triphenylphosphonium cation, cryptocyanine and mitochondria‐penetrating peptides.^11^ Therefore, mitochondria targeting is regarded as one of the efficient strategies for improving the therapeutic efficacy of phototherapies.

In addition, the imaging‐guided phototherapy has drawn extensive attention in cancer treatment since it can provide essential information such as size and location of the tumor, the optimal therapeutic time window and real‐time evaluation of treatment response, showing high potential in guiding the photoirradiation, monitoring the therapeutic process and optimizing the therapeutic efficacy.^12^ Especially, the rational integration of diverse imaging modalities to achieve multiple imaging can provide more precise information.^13^ As one of the most commonly adopted diagnostic tools in clinic, computed tomography (CT) imaging can provide 3D images to illustrate the anatomic structures around tumor region with high spatial resolutions and deep tissue penetration.^14^ However, the diagnostic accuracy of CT is limited by its low sensitivity, leading to poor contrast for soft‐tissue.^15^ Comparatively, the optical imaging such as photoacoustic (PA) and fluorescent (FL) imaging is featured with high sensitivity and noninvasiveness. PA imaging can provide functional information of biological tissues, such as blood vessel distribution and oxygenation status within tumor region.^16^ In addition, FL imaging is capable of real‐time monitoring the distribution of imaging probes within the whole body including tumor.^17^ Therefore, the combination of CT, PA, and FL imaging into one platform toward multiple imaging is expected to provide the complementary advantages/features over single‐imaging modality, facilitating the guidance and monitoring of the phototherapeutic process and outcome.

In this work, we report, for the first time, on the rational design and construction of a multifunctional nanoplatform for amplified phototherapy by concurrently achieving mitochondria‐targeting phototherapy, synergistic PTT/PDT by a single NIR irradiation (808 nm), self‐sufficient oxygen‐augmented PDT, and multiple‐imaging guidance/monitoring. Especially, perfluorooctyl bromide (PFOB) has been employed to construct PFOB nanoliposomes because PFOB is the main component of oxygen carrier, a second generation of perfluorocarbon (PFC)‐based blood substitute, showing great capacity of oxygen dissolving/delivery and excellent medium to prolong the ROS lifetime.^18^ Based on the similar oxygen storage and delivery capability of PFOB‐based nanoliposomes as compared to red blood cells (RBCs) for oxygen transportation, these PFOB‐based nanoliposomes act as the “Nano‐RBCs” for delivering oxygen into tumor tissue and subsequently amplifying the PDT efficacy.^19^ IR780 was encapsulated into PFOB nanoliposomes (designated as PFOB@LIP‐IR‐780) for achieving the PDT and PTT functionality of “Nano‐RBCs.” Compared to ICG, which had been explored as theranostic agent to significantly suppress breast cancer recently,^20^ the introduction of IR780 in this work exhibited several distinct advantages. First, the hydrophobic nature of IR780 enabled it being encapsulated into the nanoliposomes through single sonication, which was much easier than the preparation of ICG‐based nanoliposomes, contributing to better yield and encapsulation efficiency. Second, the fluorescence intensity, photostability, and ^1^O_2_ quantum yield of IR780 were much higher than those of ICG. Especially for the ^1^O_2_ quantum yield, the value of ICG was only 0.002, while the value of IR780 could reach as high as 0.127.^21^ Third, IR780 could retain preferentially at intracellular mitochondria without additional chemical conjugation of target ligands to further improve the therapeutic efficiency, which was difficult to be achieved by ICG.^22^ And based on the unique bromine (Br) atom in PFOB, it could block the X‐ray radiation and serve as a favorable CT contrast agent.^23^ Systematic in vitro and in vivo evaluations have been conducted in this work to demonstrate the high efficacy of as‐designed “Nano‐RBCs” for amplified phototherapies.

## 2 Results and Discussion

### 2.1 Design, Synthesis, and Characterization of PFOB@LIP‐IR780 “Nano‐RBCs”

**Scheme 1:**
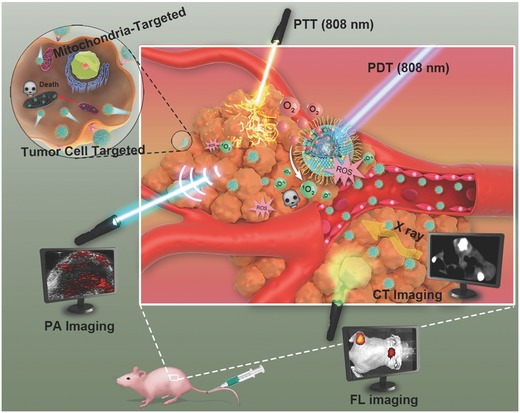
Schematic illustration of the theranostic functions of as‐synthesized NIR‐responsive “Nano‐RBCs,” including targeting cancer cells and mitochondria via loaded IR780, self‐sufficient oxygen supply by PFOB core for enhanced PDT/PTT and the guidance/monitoring by multimodal imaging including CT, PA, and FL imaging.

PFOB@LIP‐IR780, a unique NIR‐responsive “Nano‐RBCs” with core/shell structure (PFOB as the core and liposome‐PEG/IR780 as the shell) was engineered for multiple imaging‐guided phototherapy with mitochondria‐targeted property, oxygen self‐sufficient behavior and amplified synergistic PTT/PDT efficacy by a single NIR irradiation (808 nm). These nanosized PFOB@LIP‐IR780 could easily transport within the blood vessel and accumulate into tumor via the typical enhanced permeability and retention (EPR) effect (**Scheme** **1**). Upon NIR irradiation, these Nano‐RBCs can release loadings due to the damage of phospholipid bilayers by PDT generated ^1^O_2_.^24^ The PFOB core could carry the preloaded oxygen and deliver it into tumor, alleviating the tumor hypoxia and subsequently enhancing the PDT efficacy. The loaded IR780 not only acts as the photosensitizers for enhanced PDT, but also functions as PTCA for PTT. The unique photoluminescence and photothermal‐conversion capability of PFOB@LIP‐IR780 endow it with FL imaging and PA imaging performance. Especially, Br atom in PFOB could effectively block X‐ray for contrast‐enhanced CT imaging. Importantly, IR780 retains preferentially at cellular mitochondria without additional chemical conjugation of target ligands, further improving the photocytotoxicity toward target organelles during PDT/PTT process because mitochondria are highly sensitive to ROS and hyperthermia.^25^

**Figure 1:**
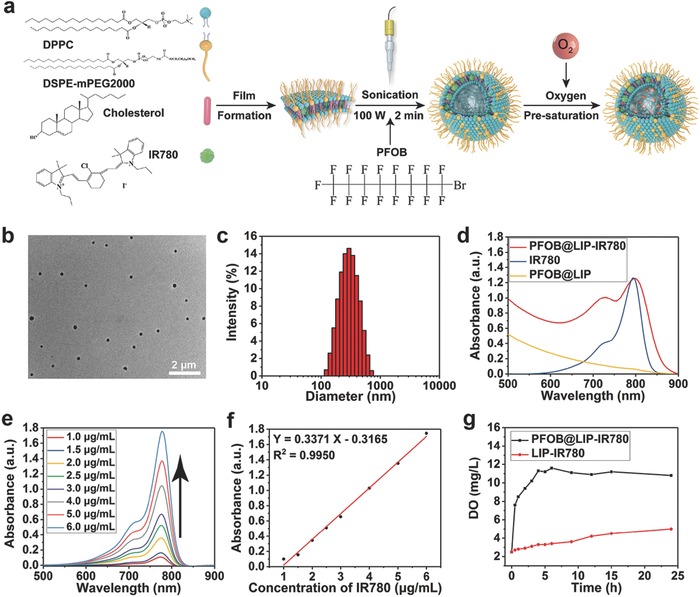
a) Schematic illustration of synthetic procedure of PFOB@LIP‐IR780. b) TEM image of PFOB@LIP‐IR780 (scale bar: 2 µm). c) Size distribution of PFOB@LIP‐IR780 as measured by DLS. d) Absorbance spectra of IR780, PFOB@LIP, PFOB@LIP‐IR780 as recorded by UV–vis–NIR spectrophotometer. e) UV–vis–NIR absorbance spectra of IR780 at elevated concentrations. f) The relative absorbance intensity of IR780 in UV–vis–NIR spectrum at the wavelength of 780 nm. g) Oxygen‐concentration changes after addition of PFOB@LIP‐IR780 and LIP‐IR780 into deoxygenated water.

PFOB@LIP‐IR‐780 “Nano‐RBCs” were synthetized via a facile one‐step emulsification method, by which lipophilic IR780 (Figure S1, Supporting Information) was incorporated into the lipid bilayer and PFOB was encapsulated in the core (**Figure** **1**a). As shown in Figure S2 (Supporting Information) and Figure 1b, the obtained PFOB@LIP‐IR‐780 was featured with highly dispersed distribution and well‐defined spherical morphology. Dynamic light scattering (DLS) measurement (Figure 1c) showed that the average size of PFOB@LIP‐IR780 was around 268.3 nm, which was in accordance with transmission electron microscope (TEM) characterization. UV–vis–NIR spectrum (Figure 1d,e) showed that PFOB@LIP‐IR780 was featured with the characteristic absorption of IR780 at 780 nm, while pure PFOB@LIP exhibited no such a characteristic absorption peak, suggesting the successful encapsulation of IR780 into the nanoliposomes. The color changed from white to green after the encapsulation of IR780 into the lipid film also confirmed the efficient loading of IR780 (Figure S3, Supporting Information). The IR780 entrapment efficiency and content were calculated to be around 92.5% and 4.4 wt%, respectively (Figure 1f). Based on the fact that perfluorocarbon‐based emulsions have been extensively employed as the artificial blood substitutes in clinic,^26^ the capacity of PFOB as oxygen reservoir was further investigated and assessed. It has been found that oxygen concentration sharply increased instantly after addition of PFOB@LIP‐IR780 with presaturated oxygen in deoxygenated water but only slightly elevated after addition of LIP‐IR780 (Figure 1g), indicating the capability of PFOB@LIP‐IR780 to store and gradually release oxygen in hypoxia environment, which is highly promising for alleviating the hypoxia of tumor and enhancing the PDT efficacy where oxygen is involved for ^1^O_2_ production.

### 2.2 Photothermal and Photodynamic Performance of PFOB@LIP‐IR780 “Nano‐RBCs”

**Figure 2:**
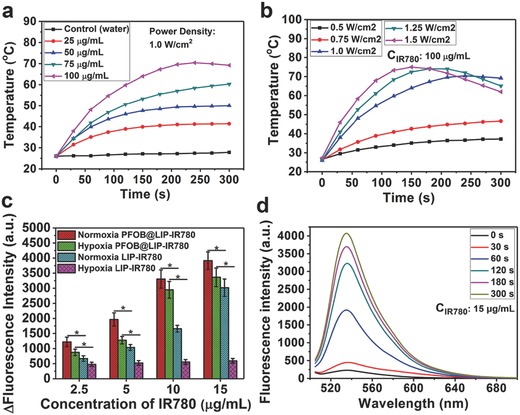
a) Temperature changes of pure water and PFOB@LIP‐IR780 aqueous suspension at different IR780 concentrations under photoirradiation (808 nm, 1.0 W cm^−2^). b) Temperature changes of PFOB@LIP‐IR780 aqueous suspension at different power densities of 808 nm laser with fixed IR780 concentration (100 µg mL^−1^). c) Concentration‐dependent ^1^O_2_ generation of PFOB@LIP‐IR780 and LIP‐IR780 under normoxic and hypoxic conditions as irradiated by 808 nm (1.0 W cm^−2^, 5 min). (Values are means ± s.d., *n* = 3, *P < 0.05.) d) Time‐dependent ^1^O_2_ generation of PFOB@LIP‐IR780 under normoxic condition as photoirradiated by 808 nm laser (1.0 W cm^−2^). The concentration of IR780 was 15 µg mL^−1^.

To evaluate the photothermal performance of PFOB@LIP‐IR780 “Nano‐RBCs,” the temperature changes of PFOB@LIP‐IR780 aqueous solution were recorded by infrared (IR) thermal imaging camera after the NIR irradiation (808 nm). Upon the 808 nm laser irradiation for 5 min at 1.0 W cm^−2^, PFOB@LIP‐IR780 solution exhibited a quick temperature increase from 25.9 to 70.4 °C at the IR780 concentration of 100 µg mL^−1^. Comparatively, the pure water used as control did not show obvious temperature elevation at the same laser irradiation condition (**Figure** **2**a and Figure S4a, Supporting Information). Furthermore, at the fixed IR780 concentration of 100 µg mL^−1^, PFOB@LIP‐IR780 “Nano‐RBCs” also followed laser power‐dependent photothermal performance with the highest temperature increment up to 75.0 °C at the power density of 1.5 W cm^−2^ for 5 min irradiation (Figure 2b and Figure S4b, Supporting Information). These results demonstrated that PFOB@LIP‐IR780 could potentially act as the photothermal nanoagent for further tumor hyperthermia.

In addition to the photothermal effect, the photodynamic property of PFOB@LIP‐IR780 was further evaluated by using typical Singlet Oxygen Sensor Green (SOSG) as the sensor to determine ^1^O_2_ production under the same laser irradiation (808 nm laser, 1.0 W cm^−2^, 5 min) both in normal and hypoxia conditions. As shown in Figure 2c, PFOB@LIP‐IR780 exhibited obvious high ROS production efficacy, which was higher as compared to that of LIP‐IR780 at normal condition. At the hypoxia condition, only small ROS amount was generated by LIP‐IR780 without the introduction of PFOB. Comparatively, the ROS generation of PFOB@LIP‐IR780 was still robust, suggesting that the oxygen‐loading ability of PFOB in the core of “Nano‐RBCs” could significantly contribute to the enhanced photodynamic effect of encapsulated IR780. At the fixed IR780 concentration of 15 µg mL^−1^, the absorption intensity of SOSG at 530 nm increased drastically with the prolonged irradiation duration (1.0 W cm^−2^), indicating the excellent ^1^O_2_ generation ability of PFOB@LIP‐IR780 (Figure 2d) and further PDT potential against cancer. The changes of SOSG peak fluorescence intensity at other concentrations of IR780 (2.5, 5, and 10 µg mL^−1^) also followed similar trend after photoirradiation for different irradiation durations (Figure S5, Supporting Information).

Due to the poor aqueous stability, fluorescence quenching, and quick elimination from the body, nanoplatforms have been designed for the delivery of IR780 and overcoming above‐mentioned deficiencies of free IR780 molecules.^27^ The photostability of IR780 was then assessed after its encapsulation into the “Nano‐RBCs” by UV–vis–NIR spectra changes of free IR780 and PFOB@LIP‐IR780 after laser exposure (Figure S6a,b, Supporting Information). As shown in Figure S6c (Supporting Information), after 2 min laser exposure, PFOB@LIP‐IR780 still maintained about 56.49% of the maximum absorbance. In contrast, free IR780 had been seriously photobleached. It has also been found that there was no obvious change of absorbance intensity of PFOB@LIP‐IR780 after stored in dark for 4 d while free IR780 almost lost its absorbance characteristics (Figure S6d–f, Supporting Information). These results demonstrated that the photostability of IR780 could be significantly improved after its encapsulation into PFOB@LIP‐IR780 “Nano‐RBCs,” which is of high significance for the following continuous in vivo imaging‐guided/monitored phototherapies.

### 2.3 In Vitro and In Vivo CT Imaging

**Figure 3:**
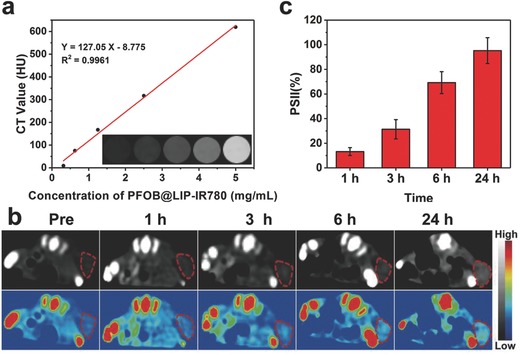
a) In vitro CT contrast images and CT values of PFOB@LIP‐IR780 at different concentrations. b) In vivo CT images of tumors on 4T1‐tumor‐bearing mice after i.v. injection of PFOB@LIP‐IR780 as recorded at different time points. The top row shows black and white images, and bottom row represents the pseudo‐colored images. c) Changes of CT‐signal intensities within tumor regions at corresponding time points. (Values are means ± s.d., *n* = 3.)

CT imaging is one of the most useful clinic diagnostic tools due to its 3D structure imaging and deep tissue penetration.^28^ Given the property of excellent X‐ray attenuation and high biocompatibility, PFOB was anticipated to be a desirable CT‐imaging contrast agent because of the presence of Br in PFOB.^23^ Therefore, the in vitro and in vivo CT contrast imaging of PFOB@LIP‐IR780 nanoliposomes were systematically investigated. It has been found that the brightness of transverse and coronal CT contrast images of PFOB@LIP‐IR780 filled in Eppendorf (EP) tubes and their corresponding pseudo‐colored signal intensities increased with the elevated concentration of the nanoliposomes (**Figure** **3**a and Figure S7a, Supporting Information), which also displayed a well‐correlated linear relationship between CT contrast and PFOB@LIP‐IR780 concentration, indicating the desirable contrast‐enhanced performance of PFOB@LIP‐IR780 for CT imaging.

Based on the desirable in vitro CT‐imaging performance of PFOB@LIP‐IR780, the in vivo contrast‐enhanced CT‐imaging performance was further assessed on tumor‐bearing mice with 4T1 breast‐cancer xenograft. As shown in Figure 3b and Figure S7b (Supporting Information), after i.v. (intravenous) injection of PFOB@LIP‐IR780 (5 mg mL^−1^, 200 µL), an obvious bright effect was observed within the tumor region, which also increased gradually over time. The pseudo‐colored images of the bright and white images were also recorded for further clearly showing the enhancement of CT imaging. The percentage of signal intensity increase (PSII) was used to analyze the CT signal intensity with prolonged time quantitatively, which reached a peak value 24 h postinjection with a clear tumor outline, demonstrating the efficient tumor accumulation of PFOB@LIP‐IR780 and further satisfactory contrast‐enhanced CT imaging (Figure 3c).

### 2.4 In Vitro and In Vivo PA Imaging

**Figure 4:**
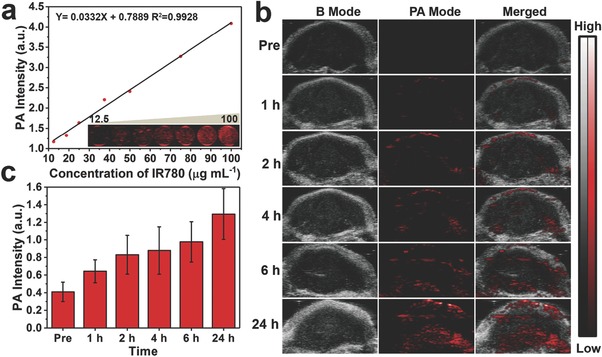
a) In vitro PA contrast images and PA values of PFOB@LIP‐IR780 at different IR780 concentrations. b) In vivo PA images of tumors in 4T1 tumor‐bearing mice after i.v. injection of PFOB@LIP‐IR780 at different time points. c) Changes of PA‐signal intensities within tumor regions at corresponding time points. (Values are means ± s.d., *n* = 3.)

PA imaging is a new imaging modality with higher sensitivity compared with CT imaging.^29^ Due to the strong absorbance in NIR region, IR780 has been used as PA contrast agent.^30^ which was also expected to show the PA‐imaging performance when it was encapsulated into PFOB@LIP‐IR780. After full spectrum scanning from 680 nm to 950 nm (interval = 5 nm) in PA imaging, it has been found that the wavelength at 780 nm was the optimal one for PFOB@LIP‐IR780 “Nano‐RBCs” enhanced PA imaging (Figure S8, Supporting Information). Therefore, the PA‐imaging performance of PFOB@LIP‐IR780 was excited and assessed at 780 nm. As depicted in **Figure** **4**a, the PA signals strengthened linearly with the increased IR780 concentration from 12.5 to 100 µg mL^−1^, suggesting that PFOB@LIP‐IR780 could effectively convert the absorbed energy into heat and trigger ultrasonic wave subsequently. After i.v. injection of PFOB@LIP‐IR780 into tumor‐bearing mice, obvious PA signal within tumor region was recorded, which also showed time‐dependent increase and reached its peak 24 h post the injection of the contrast agents (Figure 4b). In addition, the quantitative analysis of PA‐signal intensity also corresponded to the contrast‐enhanced PA imaging (Figure 4c), showing the desirable performance of PFOB@LIP‐IR780 as the PA imaging contrast agents in vivo.

### 2.5 In Vivo Biodistribution and FL Imaging

**Figure 5:**
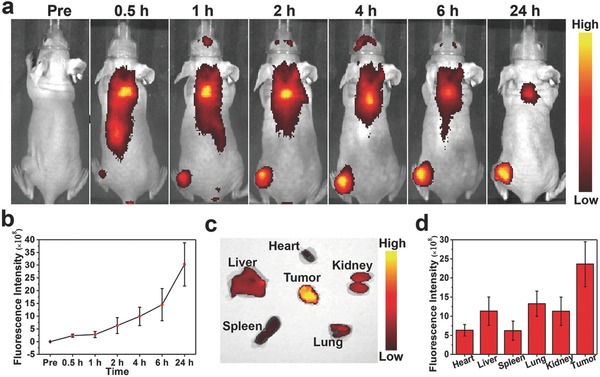
a) In vivo NIR fluorescence images of tumors in 4T1‐tumor‐bearing mice after i.v. injection of PFOB@LIP‐IR780 at different time points. b) Changes of fluorescence signal intensities within tumor regions at corresponding time points. c) Ex vivo fluorescence images of major organs and tumor dissected from mice 24 h postinjection of PFOB@LIP‐IR780. d) Quantitative biodistribution of PFOB@LIP‐IR780 in mice as determined by the average FL intensities of organs and tumors. (Values are means ± s.d., *n* = 3.)

The fluorescent property of IR780 endows PFOB@LIP‐IR780 with FL imaging performance (λ_excitation_/λ_emission_ = 745 nm/820 nm), which could be further used for the evaluation of tumor accumulation and biodistribution of these “Nano‐RBCs”.^31^ FL images were acquired at prolonged time points after i.v. injection of PFOB@LIP‐IR780. As shown in **Figure** **5**a,b, strong fluorescent signal was observed within tumor region in a time‐dependent manner and the signal in tumor region was obviously different from the normal tissue 1 h postinjection. Consistent with CT and PA imaging results, the peak value of fluorescent signals at tumor site was recorded 24 h postinjection. Therefore, the major organs and tumors were harvested for ex vivo FL imaging 24 h postinjection. It has been found that the tumor showed the highest fluorescent intensity than the other organs including heart, liver, spleen, lung and kidneys (Figure 5c,d), suggesting the effective accumulation of these PFOB@LIP‐IR780 “Nano‐RBCs” into tumor tissue.

### 2.6 Intracellular Uptake of PFOB@LIP‐IR780 and Intracellular ROS Generation by PDT

**Figure 6:**
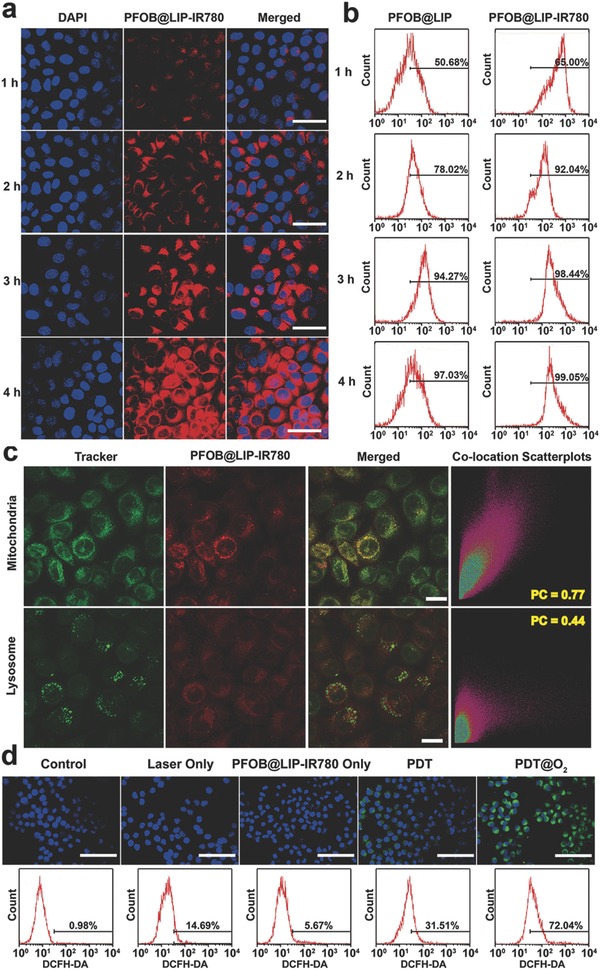
a) Intracellular uptake of PFOB@LIP‐IR780 as observed by CLSM after various intervals of incubation. The scale bars are 50 µm. b) Flow‐cytometry analysis of intracellular uptake of PFOB@LIP‐IR780 and PFOB@LIP labeled with DiI. c) PFOB@LIP‐IR780 colocalized with mitochondrial and lysosome trackers as observed by CLSM. The scale bars are 20 µm. d) Enhanced ROS production by PDT@O_2_ in 4T1 cancer cells. Confocal images (green fluorescence indicates positive staining for ROS stained with DCFH‐DA) and flow‐cytometry analysis of ROS generation by different treatments. The scale bars are 100 µm.

Efficient intracellular uptake of PFOB@LIP‐IR780 was the prerequisite to improve their phototherapeutic efficacy to kill cancer cells. Therefore, the intracellular uptake behavior of PFOB@LIP‐IR780 was then investigated by confocal microscopy. As expected, after 4 h coincubation, obvious red fluorescence of PFOB@LIP‐IR780 (labeled with DiI) was observed in 4T1 cells (**Figure** **6**a). It has been reported that IR780 exhibited preferential accumulation property toward tumor cells.^32^ From the analysis of flow cytometry results (Figure 6b), stronger red fluorescence in the cells incubated with PFOB@LIP‐IR780 was observed than cells treated with PFOB@LIP, especially 2 h postincubation, demonstrating that IR780 favored the efficient accumulation in tumor cells. Such an uptake difference between PFOB@LIP‐IR780 and PFOB@LIP narrowed gradually 3 h posttreatment when the cell endocytosis might play the main role in nanoliposome uptake and accumulation.

Then, the mitochondria‐targeting capability of PFOB@LIP‐IR780 was evaluated by comparison of subcellular localization between PFOB@LIP‐IR780 and PFOB@LIP. As shown in Figure 6c, the red fluorescence of PFOB@LIP‐IR780 overlapped well with the green fluorescence of Mito‐trakcker. In addition, its amplified confocal laser scanning microscope (CLSM) images as observed through structured illumination microscopy (SIM) clearly showed the mitochondria‐targeted accumulation of PFOB@LIP‐IR780 (Figure S9, Supporting Information). Comparatively, the poor overlap was observed in the case of Lyso‐tracker. The corresponding Pearson Correlation (PC) coefficients were determined to be 0.77 and 0.44 for mitochondria and lysosomes probes, respectively, demonstrating the relatively selective localization of PFOB@LIP‐IR780 into mitochondria. As for the PFOB@LIP (Figure S10, Supporting Information), almost no red fluorescence overlapped with green fluorescence of Mito‐trakcker (PC = 0.09), which was much lower than that of PFOB@LIP‐IR780, confirming that the presence of IR780 contributed to the mitochondrial targeting, which potentially promoted the efficacy of photoinduced cytotoxicity because mitochondria was susceptible to ROS and hyperthermia.

Motivated by desirable photodynamic performance of PFOB@LIP‐IR780 in aqueous solution, the intracellular ^1^O_2_ generation capability of PFOB@LIP‐IR780 was further assessed using 2′,7′‐dichlorofluorescin diacetate (DCFH‐DA) as the sensor, which could be rapidly oxidized by ^1^O_2_ to emit green fluorescence. As shown in Figure 6d, upon 808 nm laser irradiation (1.0 W cm^−2^) for 3 min, 4T1 cells treated with PFOB@LIP‐IR780 showed obvious green fluorescence. Comparatively, only slightly green fluorescence was observed in cells treated with LIP‐IR780, indicating the contribution of PFOB to enhancing the photodynamic effect of IR780. Flow cytometry was further used to quantitatively analyze the percentage of cells producing ROS after NIR irradiation. Cells treated with PDT@O_2_ exhibited the highest percentage of cells with ROS production (72.4%), which was much higher than the cell treated with PDT (31.5%). The cells treated with PBS and PFOB@LIP‐IR780 without laser exposure exhibited negligible fluorescence (0.98% and 5.67%, respectively). These results demonstrated that the delivery of oxygen by PFOB core in PFOB@LIP‐IR780 “Nano‐RBCs” contributed significantly to enhanced photodynamic outcome of encapsulated IR780 photosensitizer.

### 2.7 In Vitro Synergistic Therapeutic Efficacy of PFOB@LIP‐IR780 “Nano‐RBCs”

**Figure 7:**
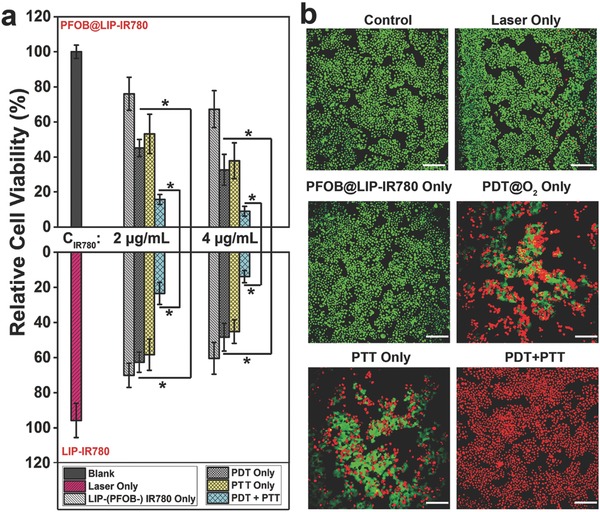
a) Relative cell viability of 4T1 cells after incubation with PFOB@LIP‐IR780 or LIP‐IR780 followed by various treatments. (Values are means ± s.d., *n* = 5, *P < 0.05.) b) Confocal images of CAM and PI costained 4T1 cells after coincubation with PFOB@LIP‐IR780 (*C*_IR780_ = 4 µg mL^−1^) for 4 h followed by various treatments. The scale bars are 50 µm.

After confirming the photothermal/photodynamic effect and mitochondrial‐targeting capability of PFOB@LIP‐IR780, its cytotoxicity against 4T1 cancer cells was evaluated by the typical CCK‐8 protocol. To verify the synergetic PTT and PDT outcome, single PTT treatment was performed by pretreating the cancer cells with vitamin C to scavenge ROS (excluding PDT effect) and single PDT treatment was conducted under ice incubation (excluding PTT effect).^33^ As shown in **Figure** **7**a, upon 808 nm laser irradiation, for the single PDT, it was notable that the photoinduced cytotoxicity of PFOB@LIP‐IR780 was higher than that of LIP‐IR780 both in normal and hypoxia conditions (Figure S11, Supporting Information), indicating that the introduction of PFOB could improve the PDT efficacy by oxygen delivery in despite of prehypoxia. When the IR780 concentration was fixed, there was no obvious difference of cell viability between PFOB@LIP‐IR780 and LIP‐IR780 group after single PTT treatment. Importantly, the combinatorial PTT and PDT effect of PFOB@LIP‐IR780 showed the highest therapeutic efficacy, which was also better than that of LIP‐IR780 because of the enhanced PDT effect.

To further evaluate the in vitro therapeutic effect of PFOB@LIP‐IR780 (IR780 concentration: 4 µg mL^−1^), calcein acetoxymethyl ester (CAM) and propidium iodide (PI) costaining was used to identify live (green fluorescence) and dead (red fluorescence) cells. As shown in Figure 7b, about 51% cell viability was detected in single PDT treatment, 30% cell viability in single PTT treatment and importantly no cell viability in synergistic PTT/PDT treatment.

### 2.8 In Vivo Detection of Tumor‐Hypoxia Status

**Figure 8:**
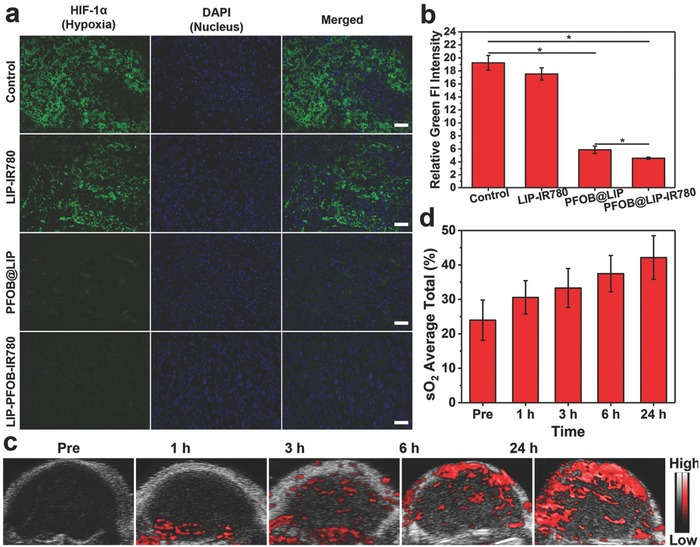
a) Immunofluorescent images of tumor slices stained by the hypoxia probe. The scale bars are 50 µm. b) Quantitative analysis of tumor hypoxia areas. c) In vivo oxyhemoglobin saturation of tumor in 4T1‐tumor‐bearing mice after i.v. injection of PFOB@LIP‐IR780 at different time points. Tumor oxygenation was detected by PA imaging in oxyhemoglobin mode. d) Quantification of oxyhemoglobin saturation at tumor sites by measuring the ratios of oxygenated hemoglobin (λ = 850 nm) and deoxygenated hemoglobin (λ = 750 nm). (Values are means ± s.d., *n* = 3, *P < 0.05.)

PFOB in “Nano‐RBCs” was used to store and deliver oxygen to overcome in vivo tumor hypoxia and improve the PDT‐therapeutic efficacy. To verify this assumption, hypoxia‐inducible factor (HIF‐1α) staining assay and oxygenated hemoglobin level detected by PA imaging system were performed to evaluate tumor hypoxic status before and after the injection of these “Nano‐RBCs.” As shown in **Figure** **8**a,b, the tumor tissue treated with PBS and LIP‐IR780 exhibited strong green fluorescence with high expression of HIF‐1α under hypoxic condition. Comparatively, for the groups treated with PFOB@LIP‐IR780 and PFOB@LIP, much weaker green immunofluorescence was detected, demonstrating the fact that tumor hypoxia was significantly alleviated by PFOB@LIP‐IR780 and a low‐level HIF‐1α expression was induced. Importantly, it has been found that HIF‐1α expression in the mice treating with PFOB@LIP‐IR780 was less than that treated with PFOB@LIP, indicating that that the tumor‐targeting nature of IR780 enabled more efficient accumulation of PFOB@LIP‐IR780 into tumor region.

To further confirm the capability of PFOB in “Nano‐RBCs” to relieve tumor hypoxia by delivering oxygen, oxygenated hemoglobin level of tumors after the injection of PFOB@LIP‐IR780 at different time points was monitored (Figure 8c,d). Oxyhemoglobin signal intensities within tumor region increased gradually with the prolonged observation time, which reached the maximum value 24 h postinjection, confirming that the presence of PFOB in PFOB@LIP‐IR780 could effectively improve oxygenation in hypoxic tumor tissues. These results demonstrated that PFOB@LIP‐IR780 could play the roles of RBCs to deliver oxygen, which then continuously supplied O_2_ to overcome the tumor hypoxia and photodynamic oxygen consumption, further amplifying the PDT efficacy at the tumor region.

### 2.9 In Vivo Synergistic Tumor Phototherapy by PFOB@LIP‐IR780 “Nano‐RBCs”

The high in vitro synergistic and amplified PTT/PDT efficacy potentially guarantees the further phototherapy of cancer by “Nano‐RBCs.” To verify this assumption, 4T1 tumor xenograft was established on nude mice for in vivo phototherapeutic assessment. When the tumor volume reached 50–80 mm^3^, mice were i.v. injected with 200 µL LIP‐IR780 or PFOB@LIP‐IR780 (5 mg mL^−1^), and the mice treated with 200 µL PBS were set as the control group. According to the biodistribution data and in vivo multiple imaging results, the time point of 24 h was adopted for laser irradiation after the injection of various agents. To demonstrate the PTT activity of PFOB@LIP‐IR780, the temperature changes of tumor site under 808 nm laser irradiation were monitored by a thermal‐imaging camera.

**Figure 9:**
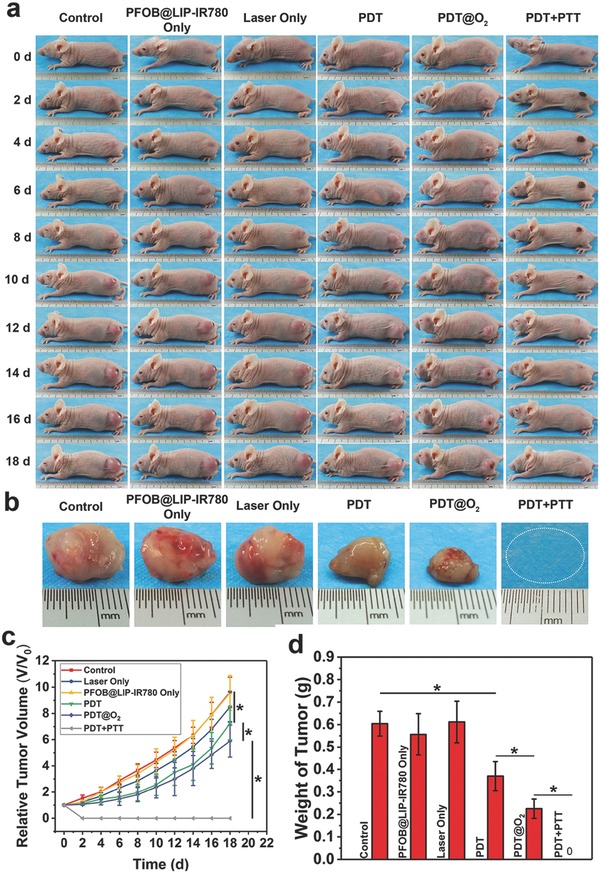
a) Photographs of 4T1 tumor‐bearing mice of six groups taken during 18 d period after various treatments. b) Photographs of tumors dissected from mice of six groups after various treatments. c) Tumor growth curves of six groups after various treatments. d) Weight of tumors 18 d post various treatments. (Values are means ± s.d., *n* = 5, *P < 0.05.)

As shown in Figure S12,b (Supporting Information), the mice treated with PDT + PTT (1.0 W cm^−2^, 10 min) exhibited obvious temperature increase from 32 °C to around 56 °C, while in other groups without laser irradiation, no significant temperature elevation was monitored. For the mice received PDT and PDT@O_2_ treatment, 30 s laser exposure followed by intervals to cool down to room temperature was performed to keep the temperature below 42 °C, which could exclude the photothermal effect (Figure S12c,d, Supporting Information).^34^ After conducting different treatments, the tumor volumes were monitored (**Figure** **9**a,b). The tumor‐volume was normalized with relative tumor volumes (*V*/*V*_0_). It has been found that the PFOB@LIP‐IR780 Only group or Laser Only group exhibited neglectable therapeutic effect on tumor growth. For the mice treated with PDT, the tumor growth was partially inhibited, showing about a 7.2‐fold increase in original tumor volume. Comparatively, for the PDT@O_2_ treatment, the tumor growth was moderately inhibited with about a 5.9‐fold increase in tumor volume, which was lower than that of tumor treated by the PDT effect as induced by LIP‐IR780. Importantly, the mice receiving combined PTT/PDT assisted by PFOB@LIP‐IR780 exhibited the complete tumor elimination, showing substantially enhanced therapeutic outcome (Figure 9c). And the weights of tumors excised from mice 18 d posttreatments also followed the similar trend as compared to tumor‐volume changes (Figure 9d).

**Figure 10:**
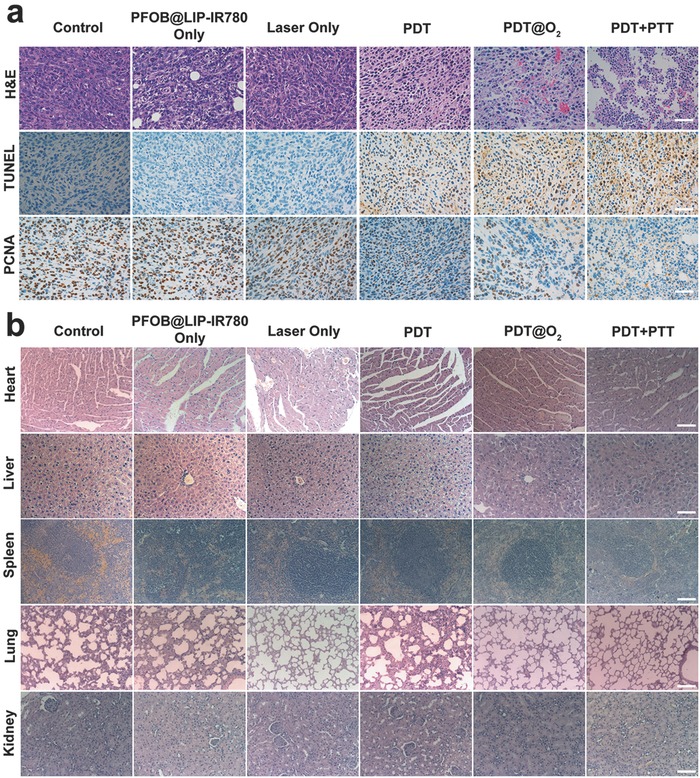
a) H&E staining, TUNEL staining, and immunochemical staining of PCNA on tumor sections from 4T1 tumor‐bearing mice after various treatments. b) H&E staining of the major organs (heart, liver, spleen, lung, and kidney) of 4T1 tumor‐bearing mice after different treatments. The scale bars are 50 µm.

Hematoxylin‐eosin (H&E), TdT‐mediated dUTP Nick‐End Labeling (TUNEL), and proliferating cell nuclear antigen (PCNA) staining on tumor sections further confirmed the synergistic and amplified PTT/PDT effect (**Figure** **10**a). As shown in H&E‐staining tumor slices, there were much more deformed nucleus (karyopyknosis, karyorrhexis, and karyolysis) in PDT/PTT group than tumor slices in other groups, indicating severe necrosis of cancer cells. In addition, the apoptosis of tumor cells in the PDT@O_2_ group was much higher than that in PDT group. The TUNEL and PCNA assay followed the similar trends. Representative apoptosis‐positive cells were indicated by dark‐brown nucleus in the TUNEL assay. Immunochemical staining of PCNA on tumor sections showed the in vivo proliferative activities of six groups where proliferative cells were stained into brown. The TUNEL and PCNA assay indicated that PDT@O_2_ was more effective than PDT, and synergistic PDT + PTT was featured with the highest therapeutic efficacy than single treatment of PDT.

To detect in vivo therapeutic biosafety of synergistic and amplified PTT/PDT against tumor as assisted by NIR‐responsive PFOB@LIP‐IR780 “Nano‐RBCs,” H&E staining of the main organs (heart, liver, spleen, lung and kidney) was performed at the end of various treatments. It has been found that no obvious histopathological lesion was observed in these organs (Figure 10b), indicating the high histocompatibility of PFOB@LIP‐IR780 “Nano‐RBCs.” The in vivo potential toxicity of the PFOB@LIP‐IR780 was further evaluated in mice during short and relatively long terms. The negligible weight fluctuations (Figure S13, Supporting Information), blood biochemical indexes (Figure S14, Supporting Information) and histopathological changes in H&E staining of the main organs (Figure S15, Supporting Information) indicated undetectable toxicity to mice at the tested dose in both short and relatively long terms, demonstrating the high therapeutic biosafety of PFOB@LIP‐IR780 “Nano‐RBCs” for phototherapies on combating cancer.

## 3 Conclusion

In summary, we have successfully constructed, for the first time, a multifunctional nanoplatform for amplified phototherapy by concurrently achieving mitochondria‐targeting phototherapy, synergistic PTT/PDT by a single NIR irradiation (808 nm), self‐sufficient oxygen‐augmented PDT and multiple‐imaging guidance/monitoring. Especially, a “Nano‐RBCs” nanosystem was designed by fabricating PFOB‐based nanoliposomes, which is the main component of oxygen carrier, a second generation of PFC‐based blood substitute. IR780 as a heptamethine dye was further integrated into the lipid shell for concurrent and synergistic PTT and PDT. These PFOB@LIP‐IR780 “Nano‐RBCs” showed the high oxygen‐delivery property for amplified PDT and mitochondria‐targeting performance for enhanced and synergistic PDT/PTT, by which the tumor could be completely eradicated without obvious reoccurrence. Importantly, based on the unique bromine atom in PFOB, excellent photoluminescence property of IR780 and desirable photothermal‐conversion performance of the nanocarrier, these PFOB@LIP‐IR780 “Nano‐RBCs” could act as the contrast agents for concurrent CT, PA, and FL multiple imaging, providing the potential imaging capability for therapeutic guidance and monitoring. The high therapeutic biosafety was also preliminarily demonstrated and systematic in vitro and in vivo evaluations have been conducted. This work paves a new way for improving the therapeutic efficacy of phototherapies by the ration combination of mitochondria targeting, synergistic PDT/PTT, self‐sufficient oxygen‐augmented PDT and multiple‐imaging guidance/monitoring.

## 4 Experimental Section

*Materials and Reagents*: IR780 iodide, PFOB, ascorbic acid (Vitamin C), and DCFH‐DA were purchased from Sigma‐Aldrich (St. Louis, MO, USA). 1,2‐dipalmitoyl‐*sn*‐glycero‐3‐phosphatidylcholine (DPPC), 1,2‐distearoyl‐*sn*‐glycero‐3‐phosphoethanolamine‐*N*‐[methoxy (polyethylene glycol)‐2000] (DSPE‐mPEG2000), and cholesterol were obtained from Avanti Polar Lipids, Inc. (Alabaster, AL, USA). 1,1′‐dioctadecyl‐3,3,3′,3′‐tetramethylindocarbocyanine perchlorate (DiI) and 2‐(4‐Amidinophenyl)‐6‐indolecarbamidinedihydrochloride (DAPI) were bought from Beyotime Technology. SOSG, MitoTracker Deep Red FM, LysoTracker Green DND‐26, and agarose were purchased from Invitrogen (Thermo Fisher Scientific). CAM, PI, and Cell Counting Kit‐8 (CCK‐8) were obtained from Dojindo (Japan). CHCl_3_ and *N*,*N*‐dimethylformamide of analytical grade were purchased from Chuandong Chemical Co. Ltd (Chongqing, China). Deionized water was obtained from a Millipore water purification system. All other reagents were of analytical grade and used as received without further purification.

*Synthesis of PFOB@LIP‐IR780*: PFOB@LIP‐IR780 was synthesized by a facile one‐step emulsion strategy. First, an appropriate mass ratio of hybrid lipid (12 mg DPPC, 4 mg DSPE‐mPEG2000, and 4 mg cholesterol) and IR780 (1 mg) was dissolved into 5 mL trichloromethane (CHCl_3_) and the mixed solution was transferred into a 100 mL round‐bottomed flask to form pure lipid or IR780‐containing films through rotary evaporation in a water bath at 55 °C. Then, 4 mL deionized water was added to rehydrated the films and the mixture was transferred to a 10 mL EP tube. Next, 0.5 mL PFOB was added to the mixture, which was sonicated using an ultrasonic probe (Sonics & Materials Inc., USA) at 100 W for 2 min in ice bath. Finally, the as‐synthesized nanoliposomes were purified by centrifugation (5000 rpm, 3 min) and stored at room temperature for further use. LIP‐IR780 was synthesized by extruding through polycarbonate membrane with pole size of 200 nm in a mini extruder. For oxygen loading, 1 mL of PFOB@LIP‐IR780 or LIP‐IR780 was placed in an aseptic oxygen chamber for 10 min to achieve oxygen saturation, by which oxygen molecules were stored into PFOB core of PFOB@LIP‐IR780 “Nano‐RBCs.”

*Characterization of PFOB@LIP‐IR780*: The morphology of PFOB@LIP‐IR780 was observed by light microscope (Olympus DP70, Canada) and TEM (Hitachi H‐7600, Japan). A laser particle size analyzer system (Nano, ZS90, Malvern instrument Ltd) was used to determine the size distribution. UV–vis–NIR absorption spectrum of IR780 (dissolved in DMSO) at different concentrations, PFOB@LIP‐IR780 and PFOB@LIP was recorded using an UV–vis–NIR spectrophotometer (UV‐3600, Shimadzu, Japan) at room temperature. The standard concentration curve of free IR780 was depicted to determinate the amount of IR780 encapsulated into liposomes at a wavelength of 780 nm. The entrapment efficiency and content were calculated by Equations (1) and (2)

(1) $$\begin{array}{l} \mathrm{Entrapment}\;\mathrm{efficiency}\;\left(\%\right)=\\ \;\;\;\;\;\frac{\left(\mathrm{Mass}\;\mathrm{of}\;\mathrm{total}\;\mathrm{IR780}-\mathrm{Mass}\;\mathrm{of}\;\mathrm{unentrapped}\;\mathrm{IR780}\right)}{\mathrm{Mass}\;\mathrm{of}\;\mathrm{total}\;\mathrm{IR780}}\;\times \;100\% \end{array}$$

(2) $$\begin{array}{l} \mathrm{Entrapment}\;\mathrm{content}\;\left(\%\right)=\\ \;\;\;\;\;\frac{\left(\mathrm{Mass}\;\mathrm{of}\;\mathrm{total}\;\mathrm{IR780}-\mathrm{Mass}\;\mathrm{of}\;\mathrm{unentrapped}\;\mathrm{IR780}\right)}{\mathrm{Mass}\;\mathrm{of}\;\mathrm{total}\;\mathrm{liposomes}}\;\times \;100\% \end{array}$$

The oxygen centration in degassed water was measured by a portable dissolved oxygen meter (YSI, 550A, Japan) after adding PFOB@LIP‐IR780 and LIP‐IR780 with presaturated oxygen.

*In Vitro Photothermal and Photodynamic Properties of PFOB@LIP‐IR780*: In vitro photothermal properties of PFOB@LIP‐IR780 were evaluated by NIR laser irradiation of PFOB@LIP‐IR780 with different IR780 concentrations (25, 50, 75, 100 µg mL^−1^) at elevated NIR irradiation power density (0.5, 0.75, 1.0, 1.25, 1.5 W cm^−2^). Water was used as the control. The temperature and infrared (IR) thermal images were recorded by an infrared thermal‐imaging camera (Fotric 226, China).

In vitro photodynamic properties of PFOB@LIP‐IR780 were evaluated by a typical SOSG assay. Briefly, 3 mL solution (PFOB@LIP‐IR780 or LIP‐IR780 at various IR780 concentrations in the solution containing SOSG at 50 × 10^−6^ m) was added into a cuvette, and irradiated with a NIR laser (808 nm) at the power density of 1.0 W cm^−2^ for different time interval. To evaluate the photodynamic effect of liposomes in hypoxia conditions, the samples were kept in a GENbox Jar which would become oxygen‐free after the color of anaerobic indicator changed from pink to colorless. The fluorescence intensity curve was recorded by a multimode reader (SpectraMax M2, Molecular Devices, USA).

*Cell Culture and 4T1 Tumor‐Bearing Mice Model*: Murine breast cancer line 4T1 cells were obtained from Shanghai Institute of Cells, Chinese Academy of Science and cultured in high glucose medium (DMEM, Gibico) supplemented with 10% fetal bovine serum (FBS) and 1% penicillin‐streptomycin solution at a humidified atmosphere of 5% CO_2_ at 37 °C. When the cultured cells reached 80% confluency, they were subcultured at a ratio of 1:3 for cell experiments and construction of tumor models. All animals (female nude mice and BALB/c mice with the weight of 17–23 g and age of 4–6 w) were purchased from the Experimental Animal Center of Chongqing Medical University. All the experiments and procedures were performed under guidelines approved by the Institutional Animal Care and Use Committee at Chongqing Medical University. To establish 4T1 tumor‐bearing mice models, 4T1 cells were suspended into serum‐free DMEM medium (1 × 10^6^ 4T1 cells in 100 µL per mouse), which were then injected subcutaneously to the flanks of the female nude mice. The volume of the tumor was calculated as [π/6 × length × (width)^2^].

*In Vitro and In Vivo CT Imaging*: CT‐imaging experiments were conducted on a clinical CT‐imaging system. PFOB@LIP‐IR780 dissolved in PBS at different concentrations (0.3125, 0.625, 1.25, 2.5, 5 mg mL^−1^) were placed in 4 mL EP tubes for in vitro CT imaging. The imaging parameters were set as follows: 200 mA, 80 kV. The CT‐signal intensities within the region of interest (ROI) were measured. For in vivo CT imaging, 4T1 tumor‐bearing mice (*n* = 3) were i.v. injected with PFOB@LIP‐IR780 saline solution (5 mg mL^−1^, 200 µL). Then, CT images were collected at different time points (pre, 1, 3, 6 and 24 h) and pseudo‐color was performed for each image by the software of Matlab (2016). The method of self‐controlled study was used to evaluate in vivo CT contrast effect. Average CT signal intensity (SI) of the tumor region of the same slice was measured. The percentage of signal intensity increase (PSII) was calculated as follows: PSII = (SI_post_ − SI_pre_)/SI_pre_ × 100%.

*In Vitro and In Vivo PA Imaging*: To evaluate the PA performance of PFOB@LIP‐IR780, a 3% (w/v) agarose gel phantom with a hole of 1 cm in diameter was initially prepared. A Vevo LAZR Photoacoustic Imaging System (VisualSonics Inc., Toronto, Canada) was used to acquire PA images. PFOB@LIP‐IR780 suspension at the IR780 concentration of 100 µg mL^−1^ was used for PA imaging at different wavelengths scanning from 680 to 970 nm (interval = 5 nm) to detect the maximum absorbance. Then, a laser at the excitation wavelength of 780 nm was used. The quantified PA signal intensity within region of interest (ROI) of each image was then analyzed by Vevo LAZR software. Different IR780 concentrations of PFOB@LIP‐IR780 dissolved in deionized water ranging from 0 to 100 µg mL^−1^ were added into the holes and then the corresponding PA images were acquired. The PA‐signal intensity in each image was measured.

For in vivo PA imaging, 4T1 tumor‐bearing mice (*n* = 3) were i.v. injected with PFOB@LIP‐IR780 saline solution (5 mg mL^−1^, 200 µL). Then, the PA images were collected at different time points (pre, 1, 2, 4, 6, and 24 h) and the average PA‐signal intensity value of the tumor regions was measured.

*In Vivo Biodistribution and FL Imaging*: For in vivo biodistribution assessment and FL imaging, 4T1 tumor‐bearing mice (*n* = 3) were i.v. injected with PFOB@LIP‐IR780 saline solution (5 mg mL^−1^, 200 µL). NIR FL images were obtained pre, 0.5, 1, 2, 4, 6 and 24 h postinjection, and the relative FL intensity of the tumor regions was measured. The major organs and tumor tissues were harvested for ex vivo imaging to detect the biodistribution of these “Nano‐RBCs.”

*Detection of Tumor Hypoxia Status*: To detect the hypoxia status of the 4T1 tumor, twelve mice were randomly divided into four groups (control, LIP‐IR780, PFOB@LIP, and PFOB@LIP‐IR780, *n* = 3 in each group). The four groups were i.v. injected with various agents (normal saline, LIP‐IR780, PFOB@LIP, and PFOB@LIP‐IR780) and the tumors were harvested 24 h postinjection for HIF‐1α staining. The green fluorescence representing hypoxia regions and relative green‐fluorescence intensity were measured with Image J. Furthermore, oxygenated hemoglobin level of tumors postinjection of PFOB@LIP‐IR780 at different time points was monitored by Vevo LAZR Photoacoustic Imaging System in oxy‐hem mode and oxyhemoglobin saturation (sO_2_ Avr Total) at tumor sites, which was recorded by measuring the ratios of oxygenated hemoglobin (λ = 850 nm) and deoxygenated hemoglobin (λ = 750 nm).

*Intracellular Uptake of PFOB@LIP‐IR780 and Mitochondrial Location*: The intracellular uptake of PFOB@LIP‐IR780 in 4T1 cells was detected by CLSM (Nikon A1, Japan). Typically, 4T1 cells (2 × 10^4^) were seeded into a laser confocal cell‐culture dish. After 24 h incubation, the culture medium was replaced with the medium containing PFOB@LIP‐IR780 (stained with DiI, λ_excitation_/λ_emission_ = 549 nm/565 nm). After different intervals incubation with PFOB@LIP‐IR780, the nucleus of 4T1 cells were stained in blue by DAPI (λ_excitation_/λ_emission_ = 364 nm/454 nm). The fluorescent images were directly recorded by CLSM. Furthermore, the quantitative intracellular uptake of PFOB@LIP‐IR780 and PFOB@LIP at different intervals was analyzed with flow cytometry.

In order to prove mitochondria selectivity of IR780, the mitochondria and lysosomes were labelled respectively to identify the localization of intracellular PFOB@LIP‐IR780 and PFOB@LIP. After 4 h incubation with PFOB@LIP‐IR780 or PFOB@LIP labeled with DiI, cells were simply incubated with MitoTracker Deep Red FM for another 30 min to label mitochondria or incubated with LysoTracker to label lysosomes. The cells were washed twice with PBS and the mitochondrial/lysosomal localization of PFOB@LIP‐IR780 and PFOB@LIP was confirmed by costaining with DiI, MitoTracker Deep Red FM (λ_excitation_/λ_emission_ = 644 nm/665 nm) or LysoTracker (λ_excitation_/λ_emission_ = 504 nm/511 nm) using CLSM and SIM. Then, the PC coefficients of each image were measured.

*Intracellular ROS Detection*: Five groups were initially divided (control, laser only, PFOB@LIP‐IR780 only, laser+LIP‐IR780 as the PDT group, and Laser+PFOB@LIP‐IR780 as the PDT@O_2_ group). The 4T1 cells were seeded with a density of 2 × 10^5^ per dish in five laser confocal cell culture dishes (or five wells of 12‐well plate for flow cytometry analysis). After incubation for 24 h, 1 mL DMEM, 1 mL PFOB@LIP‐IR780 (dispersed in DMEM), 1 mL LIP‐IR780 (dispersed in DMEM), 1 mL PFOB@LIP‐IR780 (dispersed in DMEM) were added to corresponding dish (or well). The final equivalent concentration of IR780 was 4 µg mL^−1^. The cells were further incubated for 4 h and then washed with PBS. Next, the cells were incubated with 1 mL DCFH‐DA (30 × 10^−6^ m for CLSM detection, 10 × 10^−6^ m for flow cytometry analysis) for 10 min. Subsequently, the cells were washed with PBS and irradiated by NIR laser (808 nm, 1.0 W cm^−2^) for 3 min per dish (or well) in ice bath (excluding the photothermal effect). The nucleus were stained with DAPI and the fluorescence images were observed by CLSM (or the cells were collected for flow cytometry analysis).

*In Vitro Photodynamic Therapy and Photothermal Ablation against Cancer Cells*: To test the therapeutic effects of PFOB@LIP‐IR780, 4T1 cells were seeded in a 96‐well plate (1 × 10^4^ cells per well) for 24 h. Then these 4T1 cells received various treatments including: control, laser only, PFOB@LIP‐IR780 or LIP‐IR780 only, PDT only, PTT only, and PDT+PTT. Single PTT treatment was performed by pretreating with vitamin C to scavenge ROS and single PDT treatment was conducted under ice incubation.^33^ The PFOB@LIP‐IR780 and LIP‐IR780 were diluted according to the concentration of IR780 to 2 or 4 µg mL^−1^. After 4 h coincubation, 4T1 cells were exposed to laser (1 W cm^−2^) for 5 min. Five replicates were conducted for each group. In order to further confirm that the introduction of oxygen‐loaded PFOB could enhance the PDT effect of IR780, photodynamic therapeutic effects of PFOB@LIP‐IR780 and LIP‐IR780 to 4T1 cells were also conducted at hypoxia conditions. As for the hypoxia conditions, the samples were kept in a GENbox Jar to get rid of oxygen. 4T1 cells were treated with PFOB@LIP‐IR780 and LIP‐IR780 (the concentrations of IR780 were 2 and 4 µg mL^−1^) for 4 h. Then the cells were exposed to laser (1 W cm^−2^) for 5 min and cell viabilities were tested by CCK‐8 method. Finally, the cell viabilities were determined by CCK‐8 assay. In addition, the living cells and dead cells were costained with CAM and PI solution for CLSM observation.

*In Vivo Synergistic Tumor Ablation*: For evaluating the in vivo photodynamic/photothermal efficacy of PFOB@LIP‐IR780, six groups were divided, including control, PFOB@LIP‐IR780 only, laser only, on‐off‐Laser+LIP‐IR780 as PDT group, on‐off‐laser+PFOB@LIP‐IR780 as PDT@O_2_ group, laser+PFOB@LIP‐IR780 as PDT+PTT group. Thirty 4T1 tumor‐bearing mice were involved when the tumor volume reached about 50–80 mm^3^. The mice were divided into six groups randomly (five mice per group). The first group is set as the control group, which was i.v. injected with saline solution (200 µL). The second group was PFOB@LIP‐IR780 only group i.v. injected with PFOB@LIP‐IR780 suspension (200 µL) at the concentration of 5 mg mL^−1^. The third group was the laser only group, which was i.v. injected with saline solution (200 µL) followed by 808 nm laser exposure for 10 min at the power density of 1.0 W cm^−2^. The forth group was PDT group, which was i.v. injected with LIP‐IR780 suspension (200 µL, 5 mg mL^−1^) followed by 808 nm laser (on 30 s, off to room temperature, 20 cycles) at a power density of 1.0 W cm^−2^. The fifth group was PDT@O_2_ group, which was i.v. injected with PFOB@LIP‐IR780 suspension (200 µL, 5 mg mL^−1^) followed by 808 nm laser (on 30 s, off to room temperature, 20 cycles) at a power density of 1.0 W cm^−2^. The sixth group was PDT + PTT group, injected with PFOB@LIP‐IR780 suspension (200 µL, 5 mg mL^−1^) followed by 808 nm laser exposure for 10 min at the power density of 1.0 W cm^−2^.

The temperature and IR thermal images were recorded by an infrared thermal‐imaging camera. The tumor‐volume changes of four groups were monitored with a digital camera and the weight of each mouse was recorded every other day after various treatments. The tumor‐volume changes were normalized using the relative tumor volumes *V*/*V*_0_ (*V*_0_: the initial tumor volume before the treatment). One mouse of each group was sacrificed 24 h after treatment, and the main organs (heart, liver, spleen, lung and kidney) and the tumor issues were collected and fixed in a 4% paraformaldehyde solution. Finally, the main organs were stained with H&E, and the tumor issues were stained with H&E, TUNEL, and PCNA for histopathology analysis.

*Biosatey Assay of PFOB@LIP‐IR780*: Five BALB/c mice (≈20 g, 4–6 weeks) were set as the control group and twenty BALB/c mice (≈20 g, 4–6 weeks) were randomly divided into four groups (3 d group, 7 d group, 14 d group, and 30 d group). The blood samples and major organs (heart, liver, spleen, lung and kidney) were collected after 1 d (control group and 3 d group), 7 d (7 d group), 14 d (14 d group), or 30 d (30 d group) feeding postinjection of PFOB@LIP‐IR780 (5 mg mL^−1^ 200 µL). The blood samples were collected for routine blood examination and serum biochemical index including alanine aminotransferase, aspartate transaminase, total bilirubin, creatinine, urea nitrogen, creatine kinase, and L‐lactate dehydrogenase. The major organs were stained with H&E for histological analysis.

*Statistical Analysis*: All statistical analyses were performed with SPSS 20.0 software. Data were presented as mean ± standard deviation. The significance of the data is analyzed according to a Student's *t*‐test: *P < 0.05.

## Conflict of Interest

The authors declare no conflict of interest.

## Supporting information

Supplementary
