# Correction to “Mitochondria‐Targeted Artificial “Nano‐RBCs” for Amplified Synergistic Cancer Phototherapy by a Single NIR Irradiation”

**DOI:** 10.1002/advs.75124

**Published:** 2026-03-31

Liang Zhang, Dong Wang*, Ke Yang, Danli Sheng, Bin Tan, Zhigang Wang, Haitao Ran, Hengjing Yi, Yixin Zhong, Han Lin and Yu Chen*

https://advanced.onlinelibrary.wiley.com/doi/10.1002/advs.201800049

Correction 1

In Figure 6a, the DAPI channel image corresponding to 2 h time point was inadvertently used as the DAPI channel image corresponding to the 1 h during figure assembly; however, the red channel and merged images are correct. The corrected DAPI channel image for the 1 h time point, derived from the original merged image, is provided below.


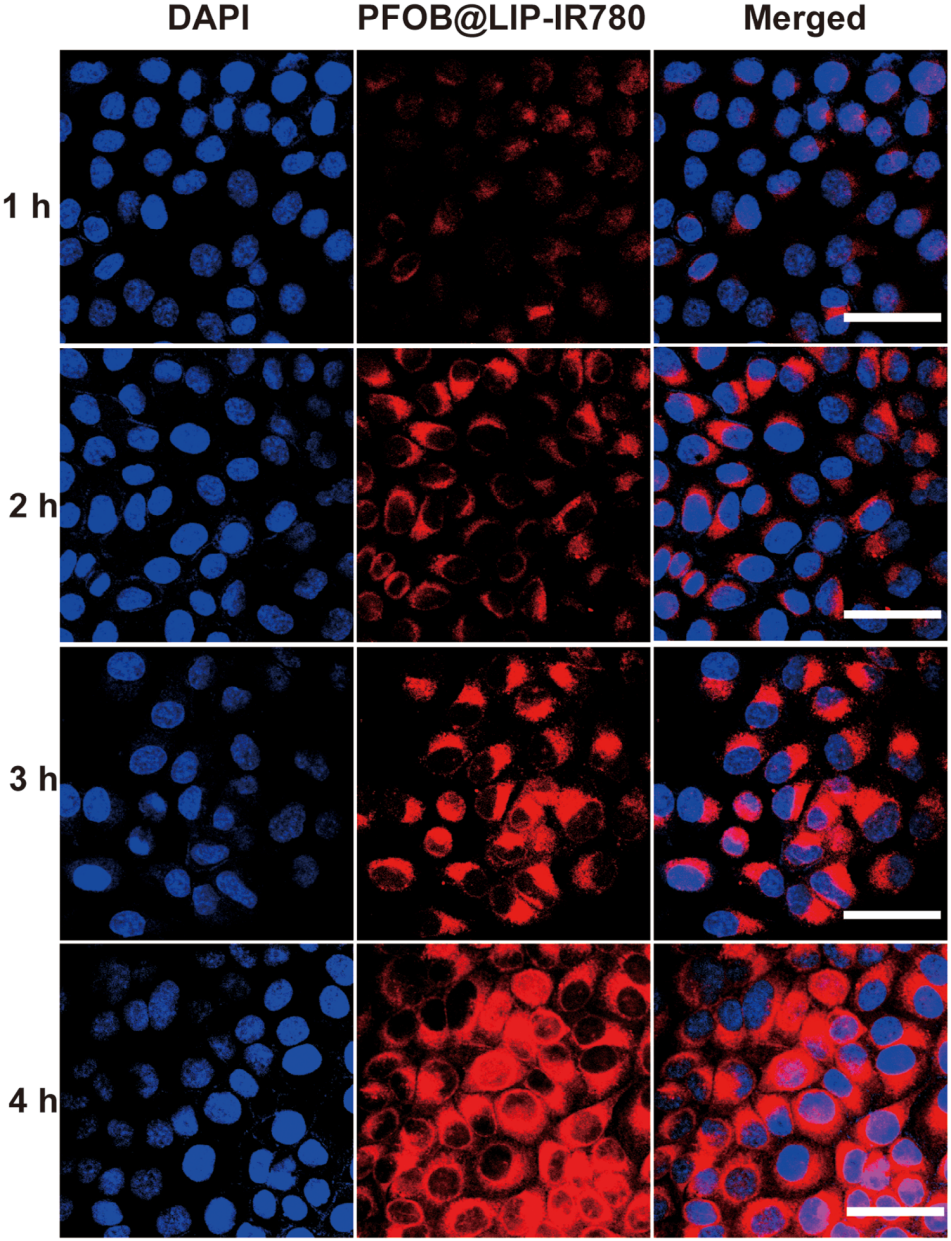


**Corrected Figure 6a**. Intracellular uptake of PFOB@LIP‐IR780 as observed by CLSM after various intervals of incubation. The scale bars are 50 µm.

Correction 2

In Figure 7b, the calcein acetoxymethyl ester (CAM) and propidium iodide (PI) co‐staining image for the PFOB@LIP‐IR780 treated group was mistakenly used. The corrected figure is provided below.


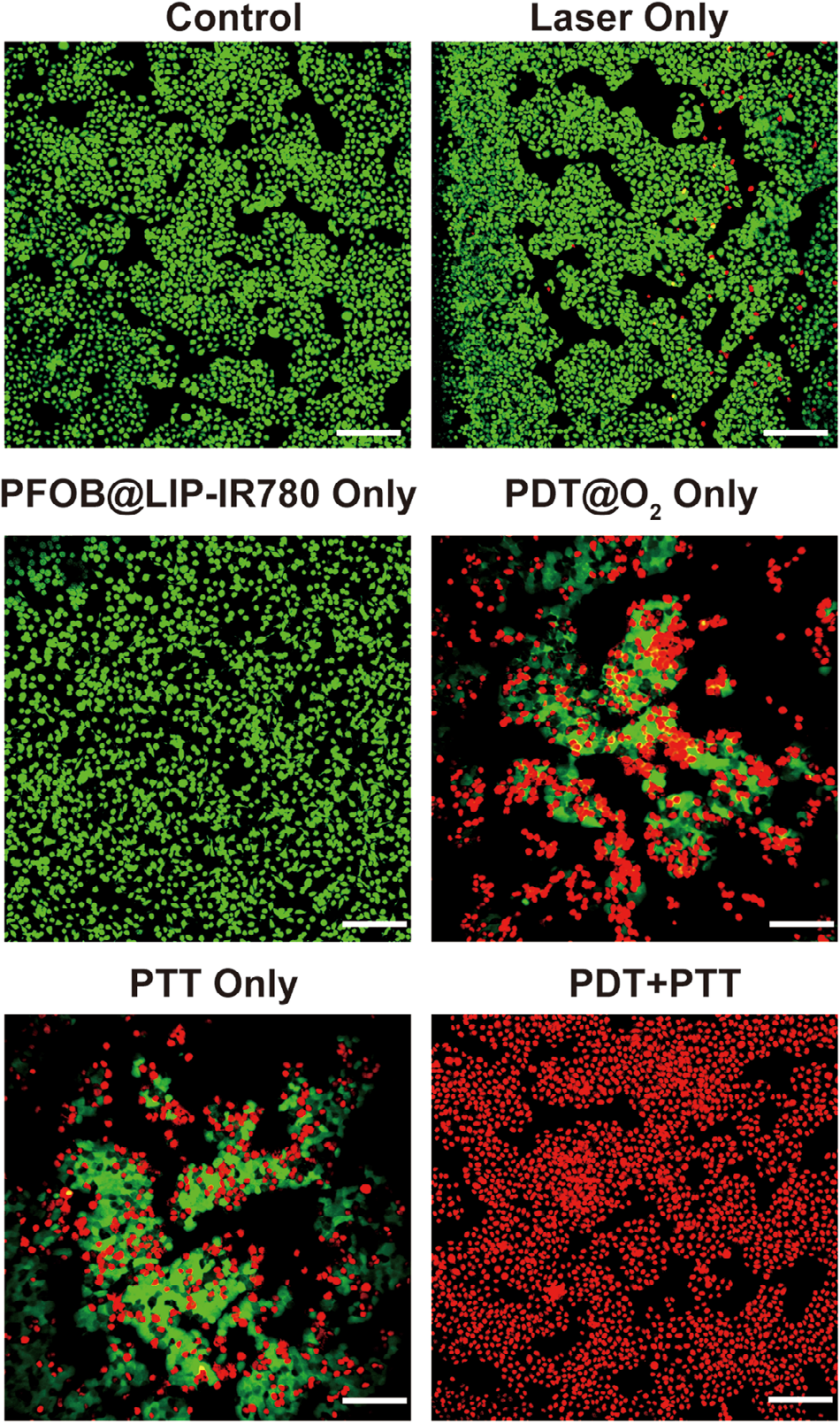


**Corrected Figure 7b**. Confocal images of CAM and PI costained 4T1 cells after coincubation with PFOB@LIP‐IR780 (C_IR780_ = 4 µg mL^−1^) for 4 h followed by various treatments. The scale bars are 200 µm.

Correction 3

In Figure 8c, the PA image corresponding to the Pre time point was mistakenly used. The corrected figure is provided below.


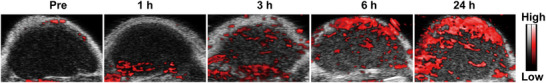


**Corrected Figure 8c**. In vivo oxyhemoglobin saturation of tumor in 4T1‐tumor‐bearing mice after i.v. injection of PFOB@LIP‐IR780 at different time points. Tumor oxygenation was detected by PA imaging in oxyhemoglobin mode.

The errors don't affect our findings and conclusions. The authors apologize for the errors and for the inconvenience it may have caused.

