# Supplementary material for: Mitochondria‐Targeted Artificial “Nano‐RBCs” for Amplified Synergistic Cancer Phototherapy by a Single NIR Irradiation
Source: Adv Sci (Weinh). 2018 May 21;5(8):1800049. doi: 10.1002/advs.201800049 (PMC6097143; doi:10.1002/advs.201800049)
Supplement: Supplementary file 1 — Supplementary [file ADVS-5-1800049-s001.pdf]

Copyright WILEY-VCH Verlag GmbH & Co. KGaA, 69469 Weinheim, Germany, 2018.

# Supporting Information

for *Adv. Sci.*, DOI: 10.1002/advs.201800049

# Mitochondria-Targeted Artificial "Nano-RBCs" for Amplified Synergistic Cancer Phototherapy by a Single NIR Irradiation

*Liang Zhang, Dong Wang,* Ke Yang, Danli Sheng, Bin Tan, Zhigang Wang, Haitao Ran, Hengjing Yi, Yixin Zhong, Han Lin, and Yu Chen**

# Supporting Information

**Mitochondria-Targeted Artificial "Nano-RBCs" for Amplified Synergistic Cancer Phototherapy by A Single NIR Irradiation**

*Liang Zhang, Dong Wang*, Ke Yang, Danli Sheng, Bin Tan, Zhigang Wang, Haitao Ran, Hengjing Yi, Yixin Zhong, Han Lin and Yu Chen**

Mr. L. Zhang, Prof. D. Wang

Department of Ultrasound, the First Affiliated Hospital of Chongqing Medical University, Chongqing, 400010, China

E-mail: wang57554@163.com

Prof. K. Yang, Prof. B. Tan

Pediatric Research Institute, Children's Hospital of Chongqing Medical University, Chongqing, 400014, China

Dr. D. Sheng, Prof. Z. Wang, Prof. H. Ran, Mr. H. Yi, Dr. Y. Zhong

Institute of Ultrasound Imaging, the Second Affiliated Hospital of Chongqing Medical University, Chongqing, 400010, China

Dr. H. Lin, Prof. Y. Chen

State Key Laboratory of High Performance Ceramics and Superfine Microstructures, Shanghai Institute of Ceramics, Chinese Academy of Sciences, Shanghai, 200050, China

E-mail: chenyu@mail.sic.ac.cn

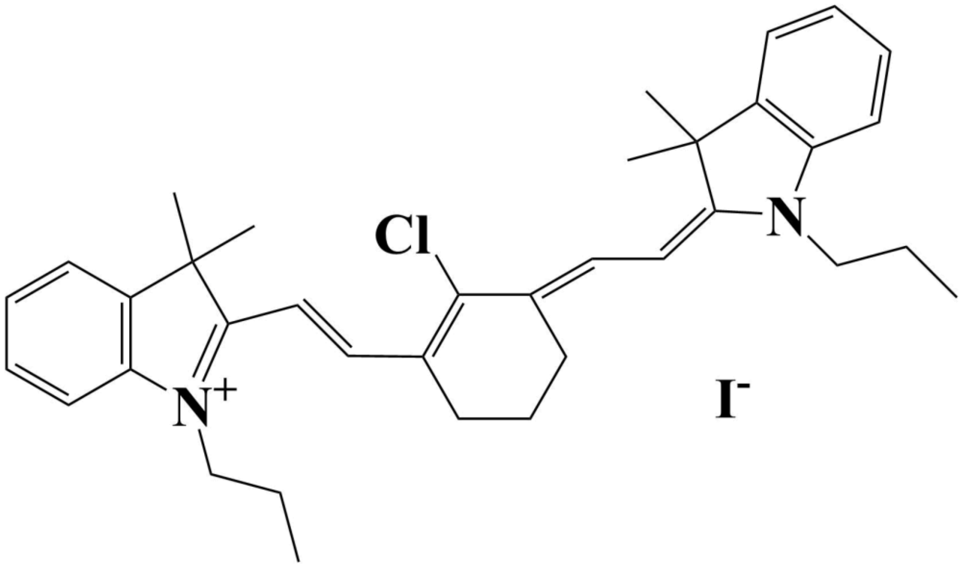


**Figure S1.** Molecular structure of IR780.

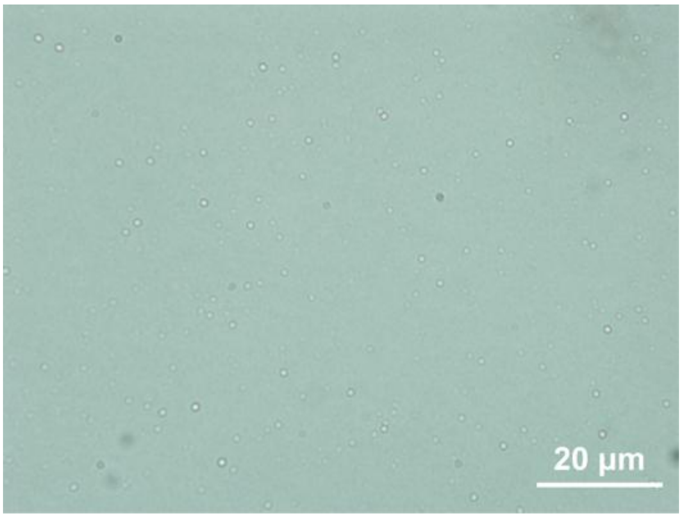


**Figure S2.** Bright-field optical image of PFOB@LIP-IR780 “Nano-RBCs” (scale bar: 20 µm).

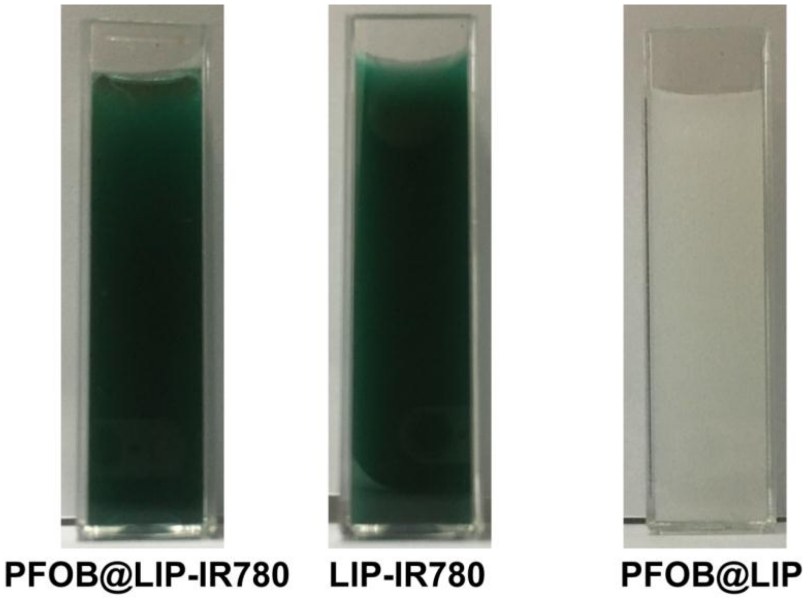


**Figure S3.** Photographs of PFOB@LIP-IR780, LIP-IR780 and PFOB@LIP dispersed in PBS.

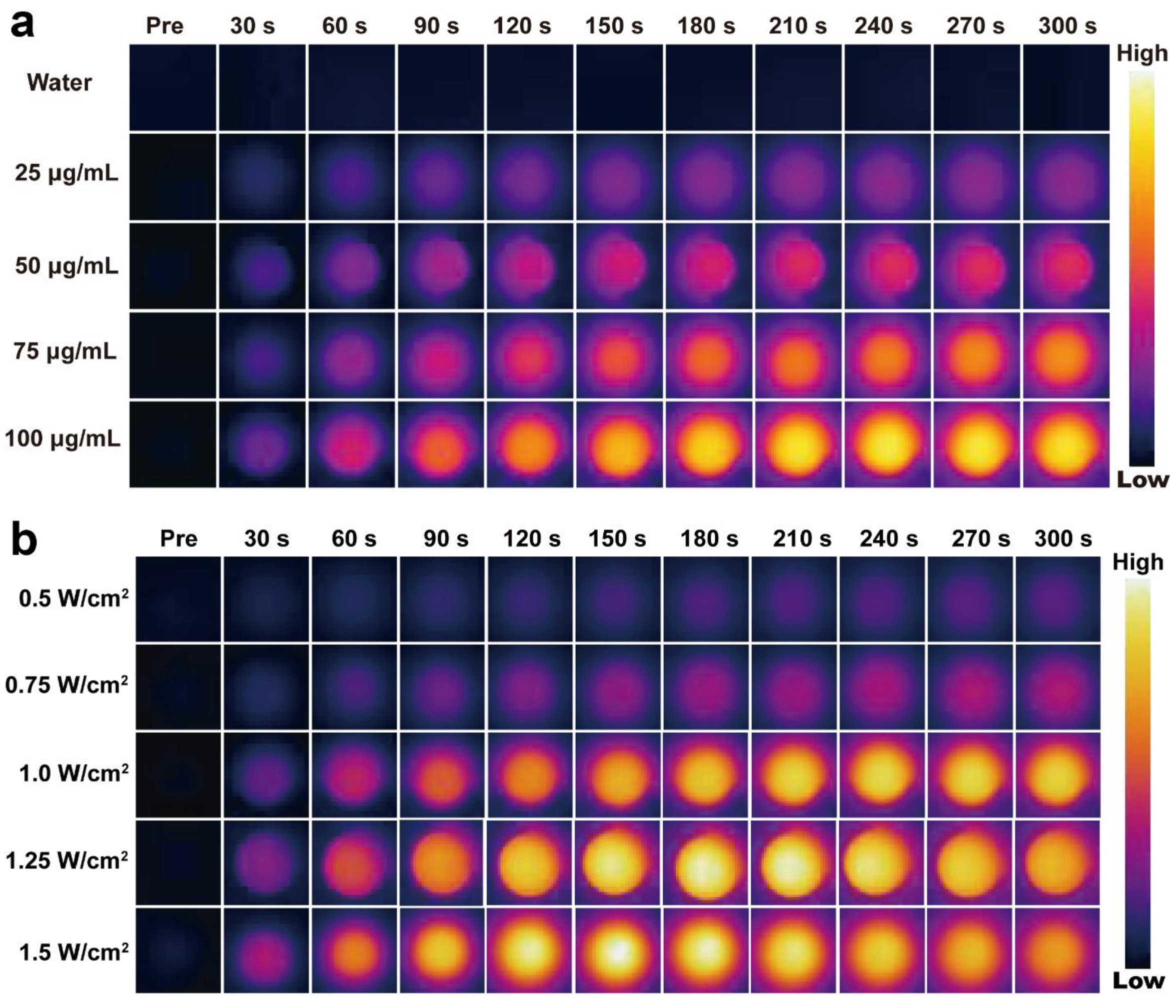


**Figure S4.** (a) IR thermal images of pure water and PFOB@LIP-IR780 aqueous suspension at different IR780 concentrations under photo-irradiation (808 nm, 1.0 W/cm$^2$). (b) IR thermal images of PFOB@LIP-IR780 aqueous suspension at different power densities of 808 nm laser with fixed IR780 concentration (100 μg/mL).

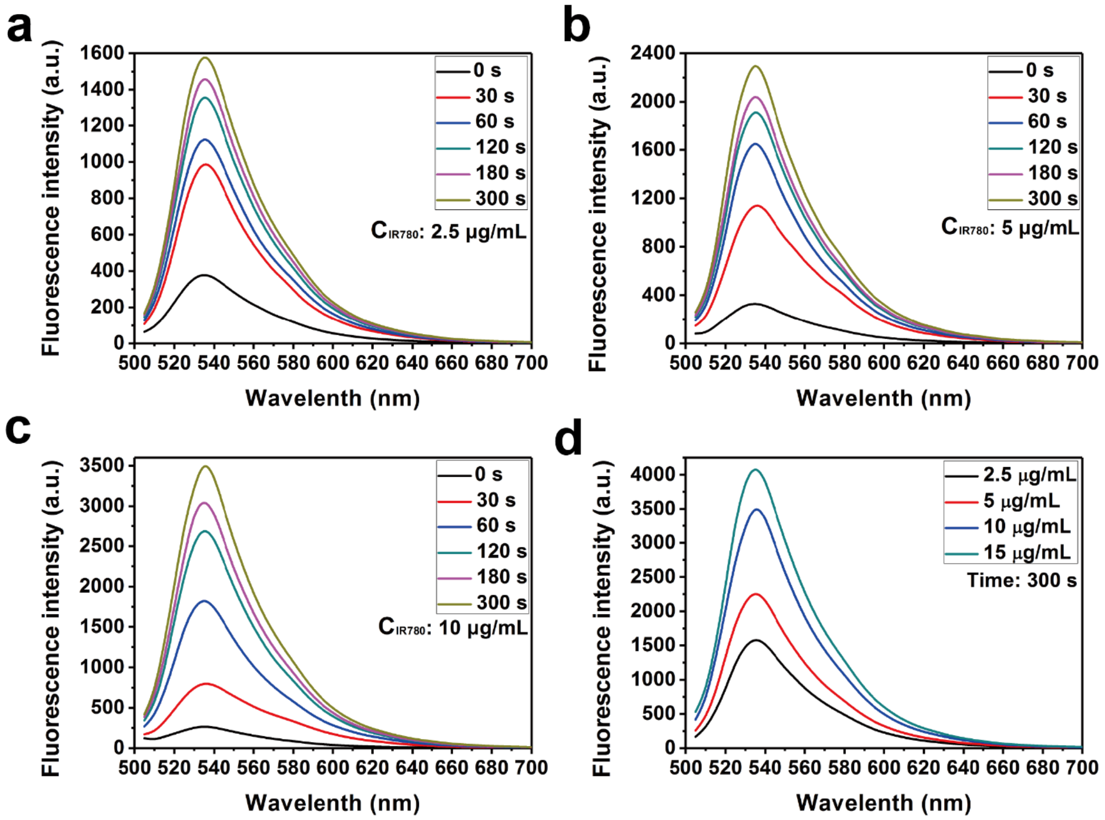


**Figure S5.** Time-dependent ROS generation of PFOB@LIP-IR780 as irradiated by 808 nm laser (1 W/cm$^2$). The concentrations of IR780 were (a) 2.5 μg/mL, (b) 5 μg/mL and (c) 10 μg/mL. (d). Concentration-dependent ROS generation of PFOB@LIP-IR780 irradiated by of 808 nm (1 W/cm$^2$, 300 s).

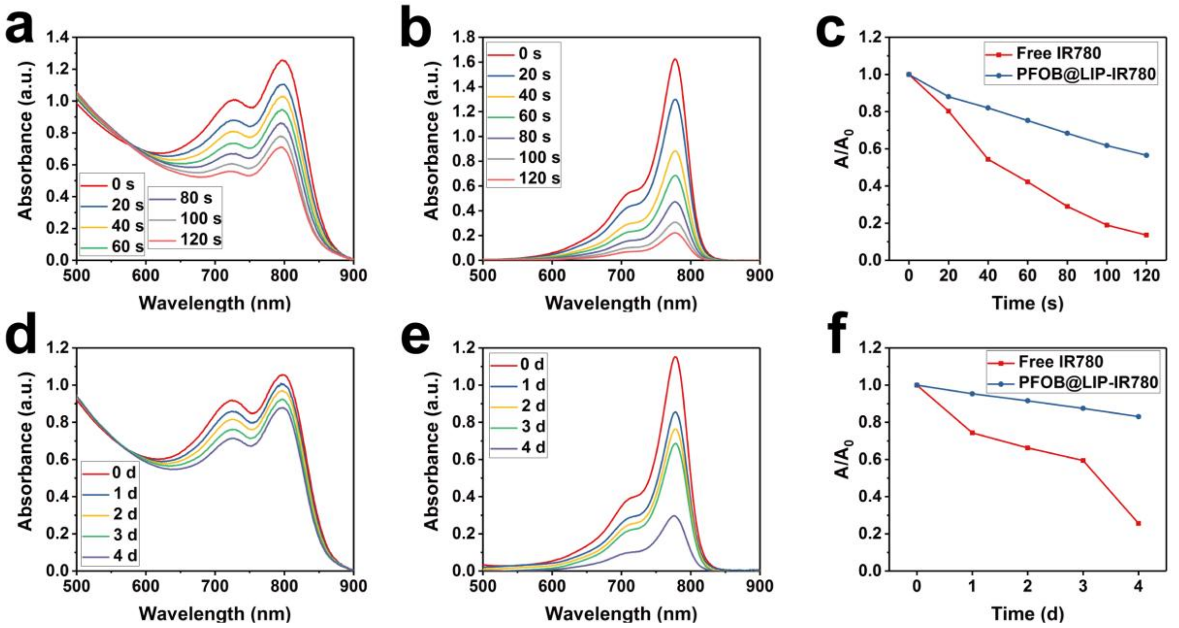


**Figure S6.** UV-vis-NIR absorption spectra of (a) PFOB@LIP-IR780, (b) free IR780 and (c) the normalized absorption after NIR laser irradiation (808 nm 1.0 W/cm$^2$). UV-vis-NIR absorption spectra of (d) PFOB@LIP-IR780, (e) free IR780 and (f) the normalized absorption for various days stored in dark.

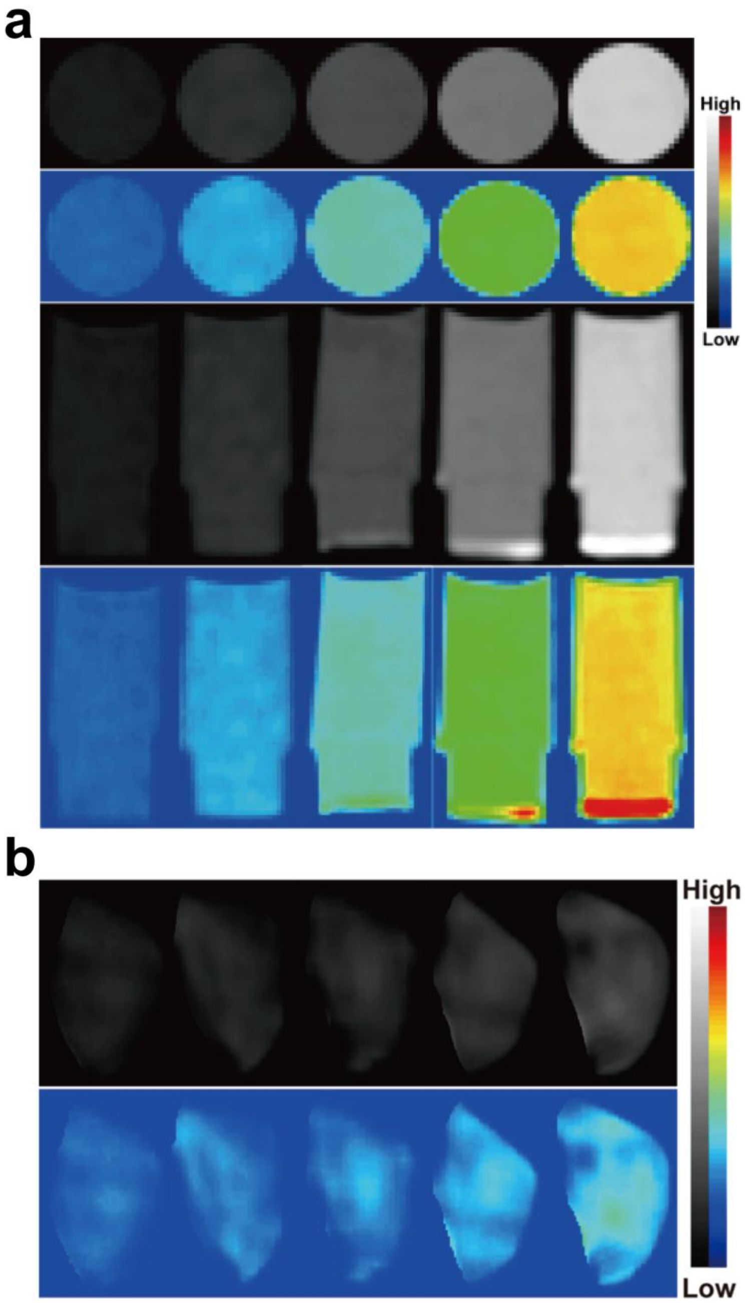


**Figure S7.** (a) CT contrast images of transverse and coronal PFOB@LIP-IR780 filled in EP tubes at different concentrations and the corresponding pseudo-colored images. (b) CT images and the corresponding pseudo-colored images of the tumor regions after *i.v.* injection of PFOB@LIP-IR780 at different time points.

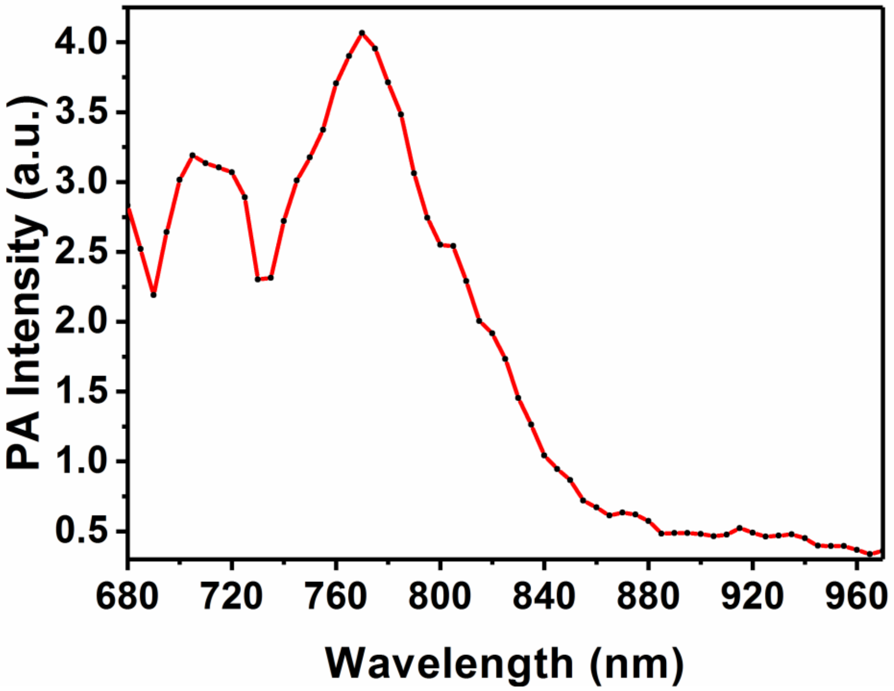


**Figure S8.** PA-signal changes of PFOB@LIP-IR780 ($C_{IR780}$ = 100 μg/mL) as irradiated by a laser at the wavelength range of 680-970 nm.

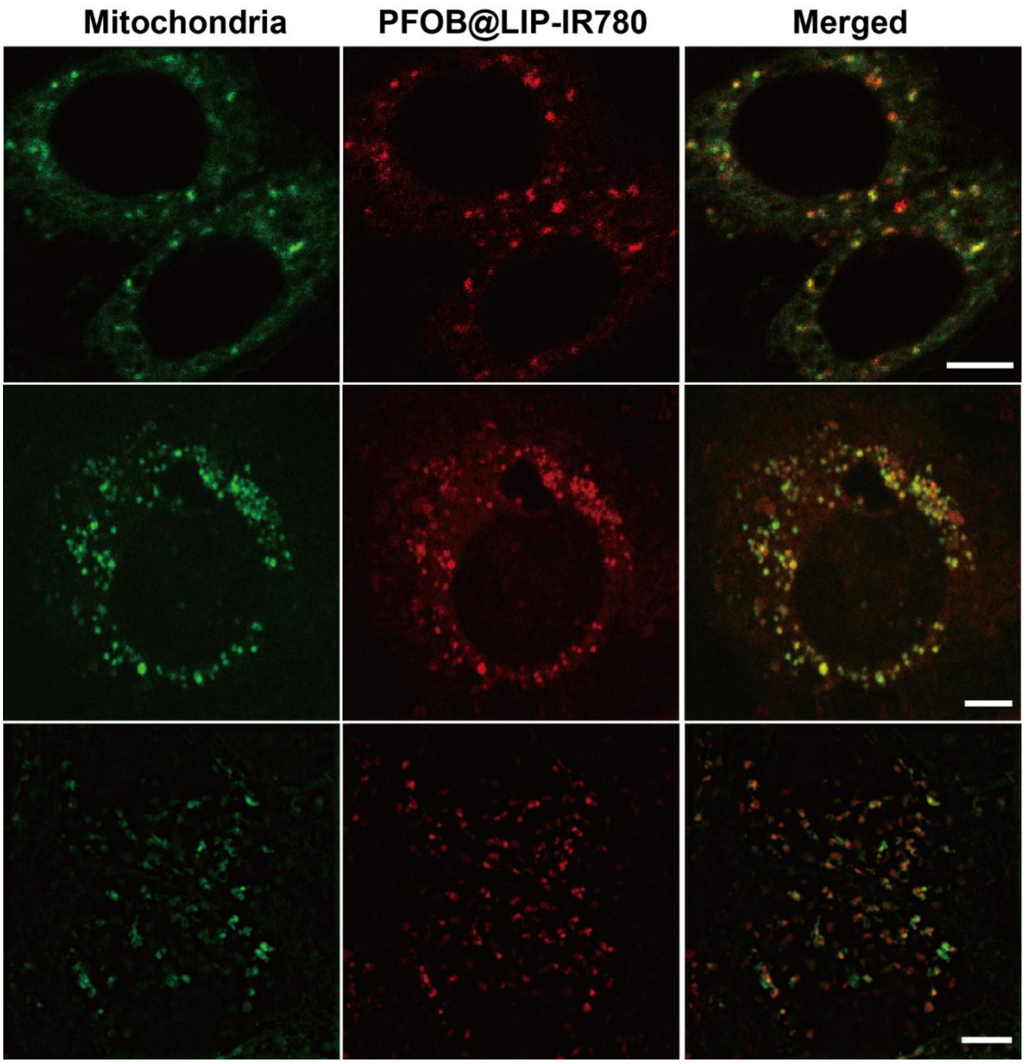


**Figure S9.** PFOB@LIP-IR780 co-localized with mitochondrial tracker as observed by CLSM and SIM. The scale bars are 5 μm.

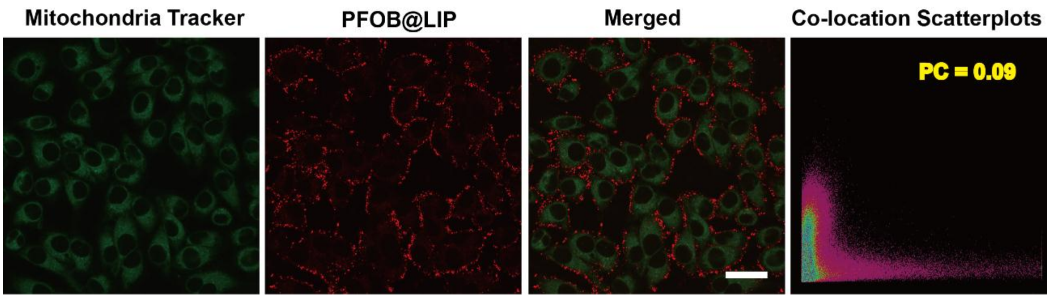


**Figure S10.** PFOB@LIP co-localized with mitochondrial trackers as observed by CLSM. The scale bars are 20 μm.

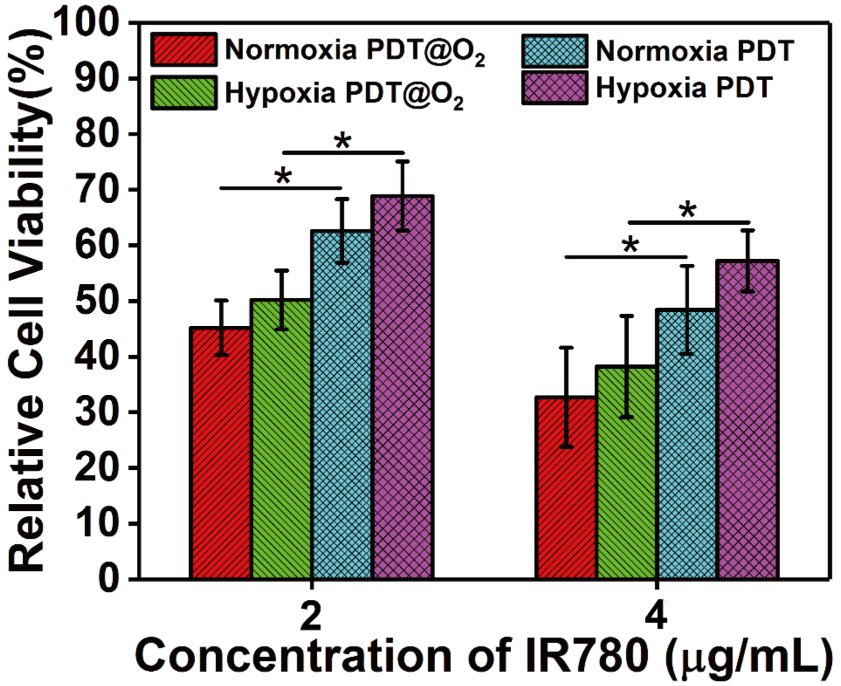


**Figure S11.** Relative cell viability of 4T1 cells after incubation with PFOB@LIP-IR780 or LIP-IR780 in normoxic and hypoxic conditions followed by PDT. (Values are means ± s.d., n = 5, *$P < 0.05$).

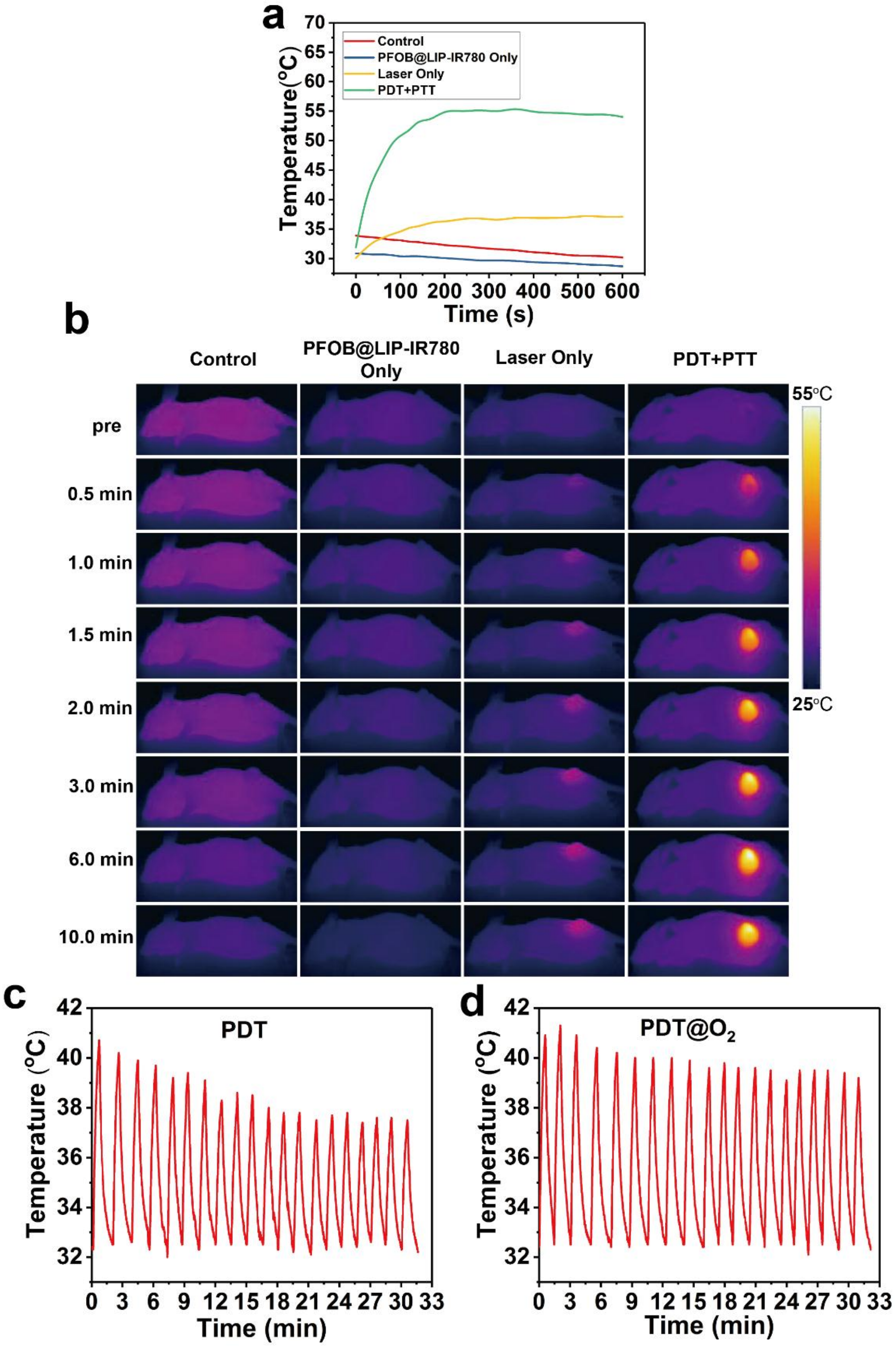


**Figure S12.** (a) Temperature changes of tumor regions and (b) the corresponding IR thermal images of the four groups (control, PFOB@LIP-IR780 only, Laser only and PDT+PTT group) as a function of irradiation duration using an 808 nm laser (1.0 W/cm$^2$, 10 min). Temperature changes of tumor regions of (c) PDT group and (d) PDT@$O_2$ group after exposure to an 808 nm laser (1.0 W/cm$^2$, on 30 s, off to room temperature, 20 cycles).

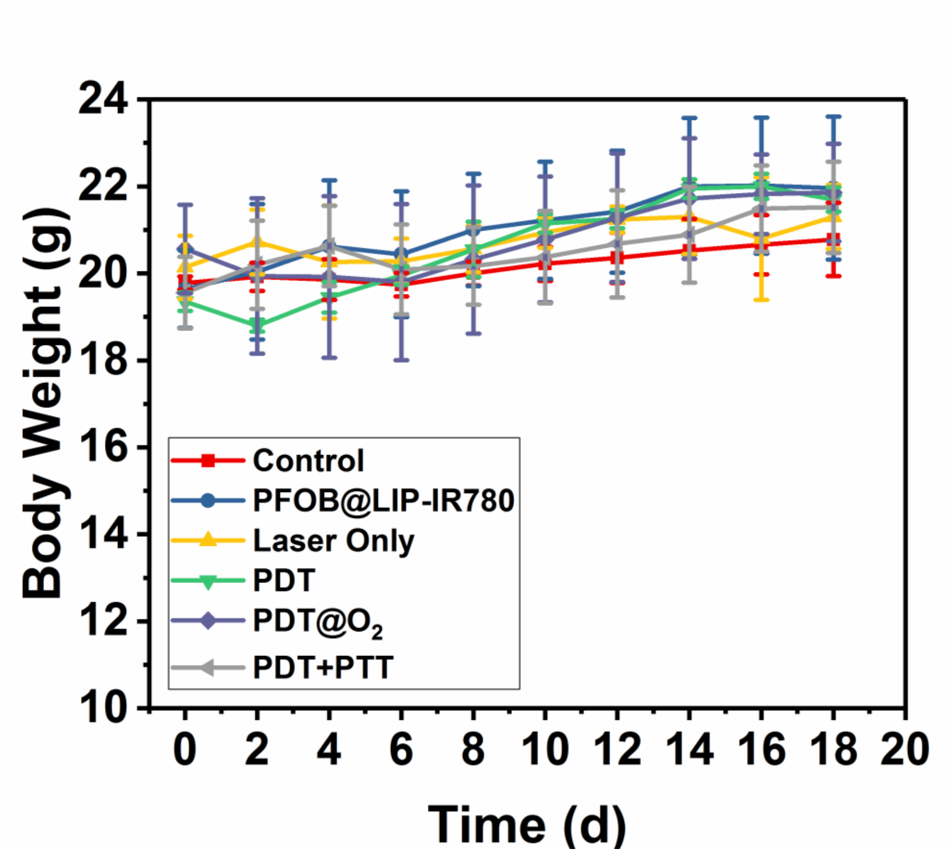


**Figure S13.** Body-weight curves (n = 5) of six groups after various treatments.

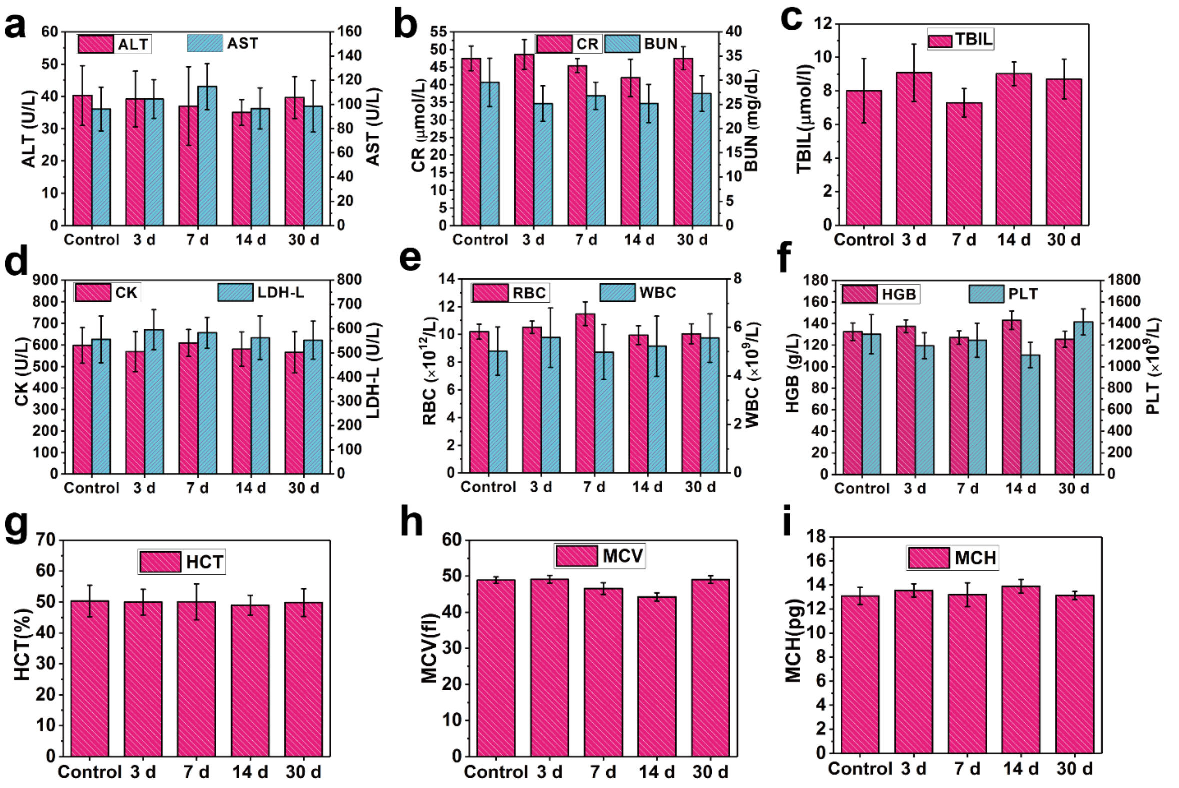


**Figure S14.** Blood biochemical indexes analysis of BALB/c mice from the control group and the experimental groups 3 ,7 14 and 30 days post intravenous injection of PFOB@LIP-IR780. (Values are means ± s.d., n = 5).

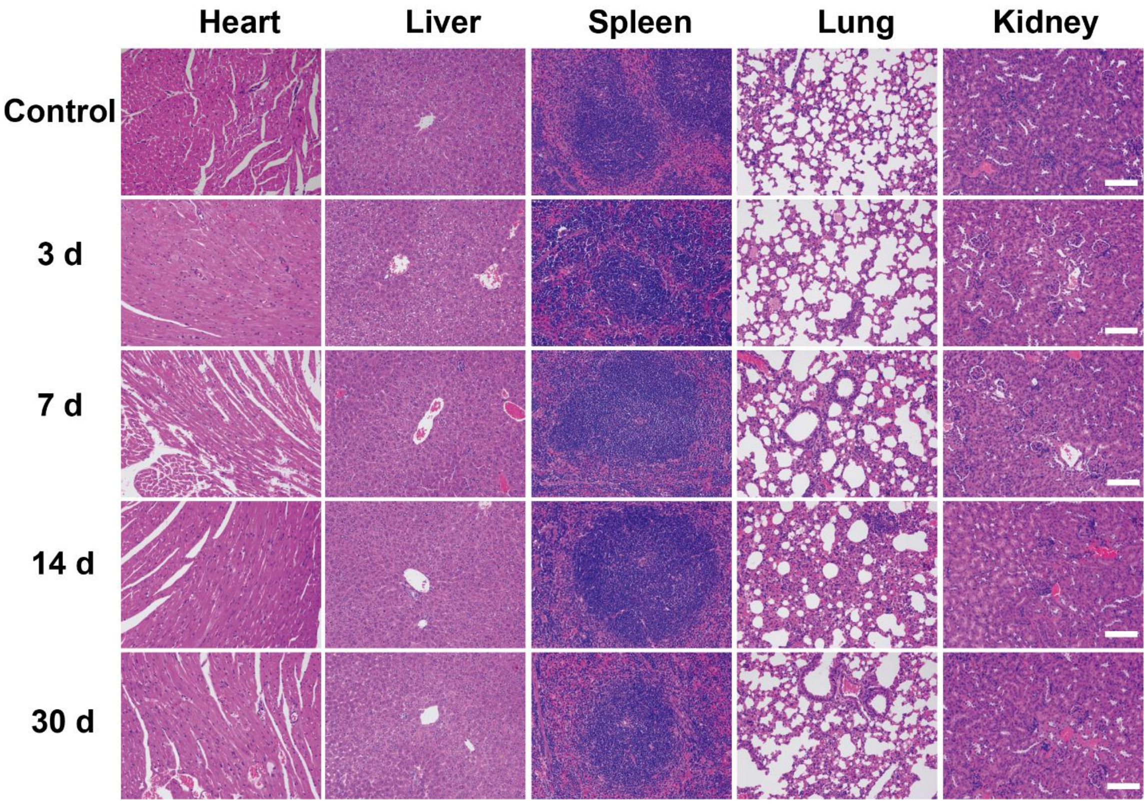


**Figure S15.** H&E staining of major organs from the control group and the experimental groups 3 ,7, 14 and 30 days post intravenous injection of PFOB@LIP-IR780. All the scale bars are 100 μm.
